# Avian auditory hair cell regeneration is accompanied by JAK/STAT-dependent expression of immune-related genes in supporting cells

**DOI:** 10.1242/dev.200113

**Published:** 2022-05-03

**Authors:** Amanda S. Janesick, Mirko Scheibinger, Nesrine Benkafadar, Sakin Kirti, Stefan Heller

**Affiliations:** ^1^ Department of Otolaryngology – Head & Neck Surgery, Stanford University School of Medicine, Palo Alto, CA 94305, USA; ^2^ Institute for Stem Cell Biology & Regenerative Medicine, Stanford University School of Medicine, Palo Alto, CA 94305, USA

**Keywords:** Basilar papilla, Hair cells, Regeneration, Single cell RNA-sequencing, Supporting cells

## Abstract

The avian hearing organ is the basilar papilla that, in sharp contrast to the mammalian cochlea, can regenerate sensory hair cells and thereby recover from deafness within weeks. The mechanisms that trigger, sustain and terminate the regenerative response *in vivo* are largely unknown. Here, we profile the changes in gene expression in the chicken basilar papilla after aminoglycoside antibiotic-induced hair cell loss using RNA-sequencing. We identified changes in gene expression of a group of immune-related genes and confirmed with single-cell RNA-sequencing that these changes occur in supporting cells. *In situ* hybridization was used to further validate these findings. We determined that the JAK/STAT signaling pathway is essential for upregulation of the damage-response genes in supporting cells during the second day after induction of hair cell loss. Four days after ototoxic damage, we identified newly regenerated, nascent auditory hair cells that express genes linked to termination of the JAK/STAT signaling response. The robust, transient expression of immune-related genes in supporting cells suggests a potential functional involvement of JAK/STAT signaling in sensory hair cell regeneration.

## Teaser

**Summary:** Gene expression changes in the facultative stem cells of the avian inner ear after ototoxic drug-induced hair cell loss *in vivo*.

## INTRODUCTION

Hearing regeneration in the avian auditory organ, known as the basilar papilla, showcases the power of non-mammalian regenerative capabilities observed in organisms such as chickens, zebrafish and salamanders. Universal regenerative signals shared between these species and their organ systems (e.g. lens, heart and limb) are thought to include nerve dependence, thrombin activation, and immunomodulation (Brockes and Kumar, 2008). Mobilization of immune cells or activation of immune genes is essential for debris removal, extracellular matrix remodeling and secretion of signals to promote proliferation and wound healing (Julier et al., 2017). The role of immune processes in regulating the pathology of hearing loss in non-regenerating mammalian systems has also gained traction: inflammation and immune cell infiltration are both seen as protective, but also harmful when linked to fibrosis after sensory hair cell loss (Hu et al., 2018; Rai et al., 2020; Raphael et al., 2007; Wood and Zuo, 2017). Although both the avian basilar papilla and the mammalian cochlea invoke an immune response after damage (Hirose et al., 2017; Warchol, 1997), one happens in the context of regeneration and the other does not. In the regenerative avian sensory epithelium, macrophage infiltration and upregulation of immune genes has been reported (Matsunaga et al., 2020; Warchol, 1997; Warchol et al., 2012).

Previous reports have investigated changes in gene expression following hair cell damage in the chicken basilar papilla and utricle (Table S1). These studies employed an *in vitro* culture model and bulk RNA-sequencing methods. *In vitro* models are valuable for immediate and homogeneous application of drugs and compounds, but are limited by the lack of surrounding tissue and the tendency of explants to lose complex organ structure. Moreover, bulk RNA-sequencing techniques lack the resolution to distinguish whether upregulated genes, such as immune genes, emanate from immune cells or otic-derived sensory epithelial cells. We recently unveiled a surgical model for local infusion of the ototoxic drug sisomicin *in vivo* via the posterior semi-circular canal of the chicken ear (Benkafadar et al., 2021; Janesick et al., 2022). This method yields rapid extrusion of hair cells and temporal synchrony of the regenerative response, which is essential for transcriptomic analyses. As this surgical method is an *in vivo* model, we are confident that it represents a relatively complete hair cell regeneration model based on the 3-week post-damage recovery data shown in the present study, as well as historical reports that deafened songbirds can relearn vocal mimicry after damage (Bermingham-McDonogh and Rubel, 2003; Ryals et al., 2013).

Leveraging recent undamaged homeostatic ‘baseline’ single cell data of the avian basilar papilla (Janesick et al., 2021), we now have a roadmap for detecting changes in gene expression occurring after ototoxic insult. We recently used the baseline dataset to explore hair cell demise from 12 to 24 h after damage (Benkafadar et al., 2021). Our present study aims to investigate the regenerative process at 30, 38 and 96 h after sisomicin infusion with single cell resolution. We characterized a group of responding supporting cells at 30 and 38 h and found the most prominent change in gene expression in these cells was the upregulation of immune-related genes linked to JAK/STAT signaling. We further show that the upregulated genes within the supporting cell layer are linked to JAK activation and that inhibition of JAK/STAT signaling abolishes the upregulation of these genes. We identify distinct markers that are expressed in responding supporting cells as well as in newly regenerated nascent hair cells, including *USP18* which we posit to halt the JAK/STAT signaling response, and *CALB2*, which was not previously appreciated as an early marker of the regenerative response. *In situ* hybridization reveals that immune-related genes are predominantly expressed on the medial side of the epithelium. Collectively, our results identify JAK-STAT activation-dependent expression of immune-related genes in supporting cells followed by differentiation into nascent, regenerated hair cells.

## RESULTS

### Immune-related genes are expressed in the chicken basilar papilla after hair cell loss *in vivo*

We recently developed a surgical method to eliminate all hair cells in the chicken basilar papilla by infusing the ototoxic aminoglycoside sisomicin into the inner ear via canalostomy (Benkafadar et al., 2021; Janesick et al., 2022). This damage paradigm differs from existing models because it requires only a single application of the ototoxin, and hair cell extrusion happens within 24 h (Benkafadar et al., 2021; Janesick et al., 2021), resulting in temporal synchrony of the regenerative response. Forty-eight hours after damage, we dissected the basilar papilla and employed a rapid ‘cold peeling’ method for obtaining pure sensory epithelia, which does not require the application of proteases and time-consuming incubation (Janesick et al., 2021, 2022). This method allows epithelial cells to be harvested and lysed within 10 min of sacrificing the animal. We infused sisomicin into the left inner ears of 7-day-old chickens (P7) and isolated RNA 48 h post-surgery (P9) from cold-peeled contralateral control and damaged epithelia. Triplicate samples were submitted for bulk RNA-sequencing (RNA-seq).

**Fig. 1.**
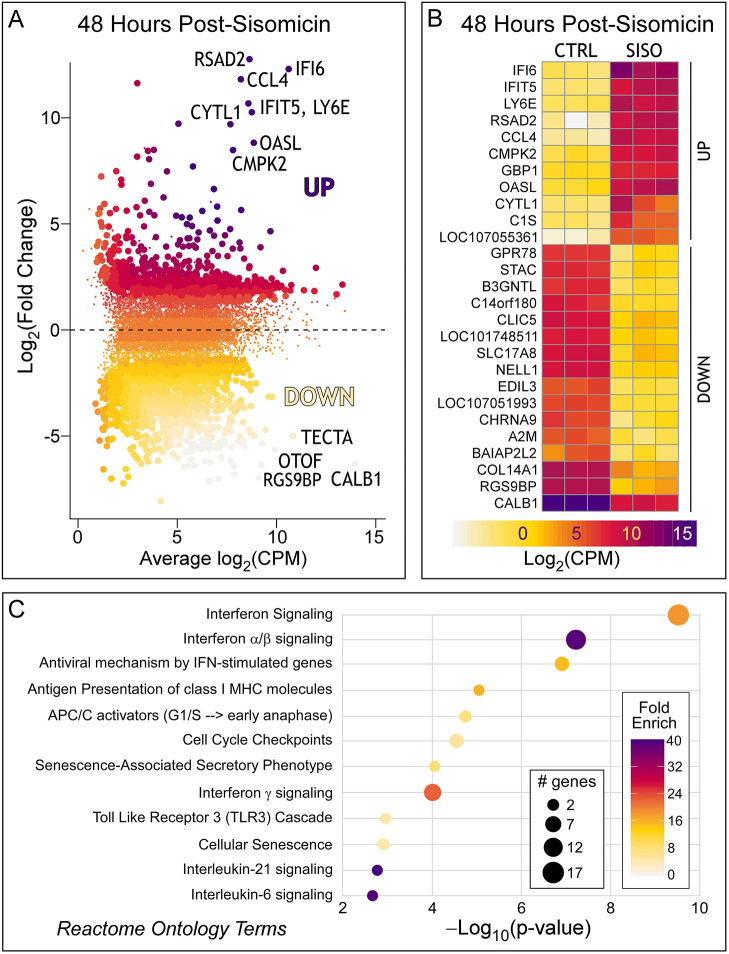
**Differentially expressed genes and their ontology 48 h post-sisomicin treatment.** (A) Mean-difference volcano plot highlighting genes with log_2_(fold change)>2 and FDR<0.05 (larger dots) (for an interactive plot, see https://umgear.org/p?l=5d177e1c&g). Fold change is relative to the contralateral control ear. CPM, counts per million reads. (B) Heatmap showing all three biological replicates per treatment, highlighting genes that meet the criteria of log_2_(fold change)>2, FDR<0.05 and log_2_CPM>5. CTRL, control (undamaged); SISO, sisomicin damaged; FDR, false discovery rate (Benjamini-Hochberg-corrected *P*-value). (C) Gene ontology analysis of sisomicin upregulated genes using the pathfindR analysis tool and Reactome database. See Table S2 for the full table of genes, their counts, differential expression and associated FDR.

RNA-seq reads were annotated and yielded 96.9% uniquely mapped reads and 22,758 genes. Cells from control and sisomicin-infused basilar papillae clustered into distinct groups and correlated well between the two conditions (Fig. S1A). The data were normalized and filtered (Rau et al., 2013; Robinson and Oshlack, 2010) to remove constant and low-abundance transcripts, leaving 11,952 genes (Fig. S1B-D). Differential expression analysis with EdgeR (Robinson et al., 2010) revealed 637 genes with log_2_FC≥2 in the sisomicin-infused condition and FDR<0.05, relative to the control, where FC is fold-change and FDR is false discovery rate (Fig. 1A). The entire differential expression dataset can be found in Table S2 and visualized in gEAR (https://umgear.org/p?l=5d177e1c&g). 995 genes were expressed at log_2_FC<−2 in the sisomicin-infused condition relative to control. As hair cells are missing in the damaged tissue, we expected that the majority of genes expressed at log_2_FC<−2 would account for missing hair cell genes. We created a heatmap for the top differentially expressed genes of log_2_FC>5, FDR<0.05 and log_2_CPM>5, where CPM is counts per million (Fig. 1B). As expected, most of the lowest expressed genes are hair cell specific, many of which were recently validated by *in situ* hybridization (Janesick et al., 2021). Three exceptions are *A2M* and *COL14A1* (supporting cell genes), and *CALB1* (expressed in hair cells and supporting cells). The downregulation of hair cell genes serves as an internal validation, confirming that the sisomicin treatment had eliminated auditory hair cells at 48 h post-surgery. We previously verified that the number of supporting cells remained unchanged after damage (Benkafadar et al., 2021); however, *TECTA*, a gene encoding a structural protein of the tectorial membrane, was significantly downregulated in supporting cells.

Gene ontology and Reactome analysis (Jassal et al., 2019; Ulgen et al., 2019) identified a strong correlation between top upregulated genes and interferon signaling (Fig. 1C). Interferon response genes are often activated via the JAK/STAT signaling pathway and are typically associated with macrophages and neutrophils (Goossens et al., 2013; Santhakumar et al., 2017). Although their nomenclature (IFI=‘interferon inducible’) suggests a causal connection to interferon signaling, these genes are not necessarily induced by interferon ligands but by a variety of other cytokines that generally signal canonically via the JAK/STAT pathway. To avoid confusion about the yet-to-be-determined source of the inducing signaling factor, we call the upregulated group of genes ‘immune-related’. There is evidence that macrophages infiltrate the chicken basilar papilla after ototoxic insult (Warchol et al., 2012). However, chemically or genetically mediated macrophage depletion does not inhibit regeneration of hair cells in the chicken basilar papilla (Warchol et al., 2012) or the zebrafish lateral line (Warchol et al., 2021). A limitation of the bulk RNA-seq approach is that it cannot rule out the possibility that there is infiltration of blood and immune cells into the basilar papilla after sisomicin infusion, which could explain the upregulation of immune-related genes. We hypothesized that single-cell RNA-seq would provide the required cellular resolution to determine the specific cell type(s) in the basilar papilla that express the identified upregulated genes after sisomicin-induced hair cell loss.

### Single-cell RNA-sequencing of the regenerating chicken basilar papilla

Pure sensory epithelia were dissociated into individual cells and processed for single-cell RNA-seq as previously described (Ellwanger et al., 2018; Janesick et al., 2021). In total, we analyzed 1891 cells at five different time points, each representing three independent clutches of chickens (Fig. S2). We chose the 30- and 38-h timepoints for analysis because this is the peak time post-sisomicin infusion for entry of supporting cells into S phase, and hence is affiliated with proliferation of supporting cells (Janesick et al., 2022). Ninety-six hours post-sisomicin, supporting cells no longer enter S phase, and the first regenerated MYO7A-positive new hair cells are detectable with immunohistochemistry (Janesick et al., 2022). Our single-cell sequencing libraries captured 17,557 expressed genes. Data quality control flagged 3987 low-abundance genes and 670 information-poor cells, which were removed from downstream analyses. The remaining 1221 cells were normalized with SCnorm (Bacher et al., 2017) and log_2_-transformed (see Materials and Methods section).

**Fig. 2.**
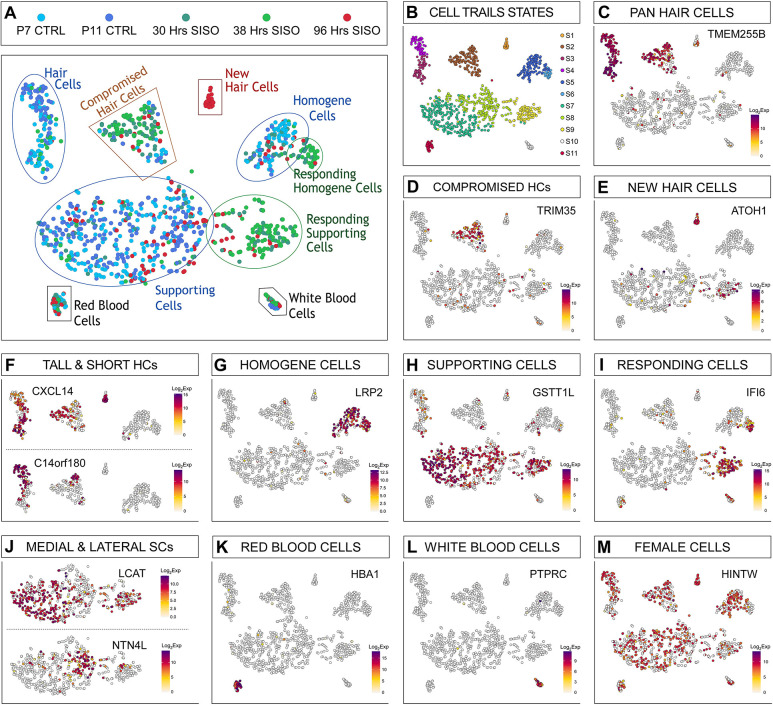
**Single cell RNA-sequencing of the regenerating chicken basilar papilla.** T-distributed stochastic neighbor embedding (tSNE) plots of all profiled cells representing five timepoints, clustered with CellTrails. (A) Seven major epithelial cell groups are outlined. CTRL, control (undamaged); SISO, sisomicin damaged. (B) CellTrails clustering reveals 11 states. (C,F-H,J) All baseline-supporting cell, hair cell and homogene groups were identified by markers from Janesick et al. (2021). HCs, hair cells; SCs, supporting cells. (D) Compromised hair cells marked by *TRIM35* were defined by Benkafadar et al. (2021). (E) New hair cells express *ATOH1*. (F) *CXCL14* is a tall hair cell marker and *C14orf180* is a short hair cell marker. (G) LRP2 is a homogene cell marker. (H) GSTT1L is a supporting cell marker. (I) Responding supporting cells express *IFI6*, which was identified in the bulk RNA-seq analysis (see Fig. 1 and Table S2). (J) *LCAT* is a medial (i.e. neural) and *NTN4L* is a lateral (i.e. abneural) supporting cell marker. (K,L) Hematocytes. (M) *HINTW* tracks female cells. Scale bar: log_2_ transformed, normalized expression counts.

Dimensionality reduction using CellTrails (Ellwanger et al., 2018) revealed seven major epithelial cell groups (Fig. 2A) and 11 clusters (Fig. 2B). Hair cells were identified by the marker *TMEM255B* (Fig. 2C; Janesick et al., 2021). At the 30- and 38-h timepoints, we observed compromised hair cells that expressed *TRIM35* (Fig. 2D; Benkafadar et al., 2021). These hair cells were also *TMC2*-high and *TMC1*-low (Fig. S3), suggesting that they were derived from the distal end of the basilar papilla, where the least-sensitive hair cells to sisomicin damage are located (Janesick et al., 2022). *TRIM35*-positive distal hair cells are likely to mount a repair response and represent a group of hair cells that survived the sisomicin treatment. At 96 h, *ATOH1*-positive hair cells segregated distinctly (Fig. 2E) – we classified these as newly regenerated hair cells. Subclusters of tall and short hair cells were identified by marker genes *CXCL14* and *C14orf180* (Fig. 2F), respectively, explored in detail in Janesick et al. (2021). Homogene and supporting cells were distinct populations marked by *LRP2* and *GSTT1 L*, respectively (Fig. 2G,H; Janesick et al., 2021). 30- and 38-h post-sisomicin cells harbored distinct groups of responding supporting cells and homogene cells (Fig. 2I). Leveraging knowledge from the bulk RNA-seq data (Fig. 1, Table S2), the responding supporting and homogene cells expressed *IFI6*, a gene first identified as induced by interferon (Tahara et al., 2005) (Fig. 2I). Subclusters of medial and lateral supporting cells were identified by marker genes *LCAT* and *NTN4L* (Fig. 2J; Janesick et al., 2021). Red and white blood cells were identified by *HBA1* and *PTPRC*, respectively (Fig. 2K,L). Male- and female-derived cells were present in approximately equal numbers (♀=586; ♂=635; Fig. 2M), as indicated by the ubiquitous female transcript derived from the *HINTW* gene (Nagai et al., 2014). Annotation for the cell types within each timepoint was visualized with pie charts in Fig. S2 and quantified in Table S3.

### Immune-related genes are robustly upregulated in supporting cells after hair cell damage

**Fig. 3.**
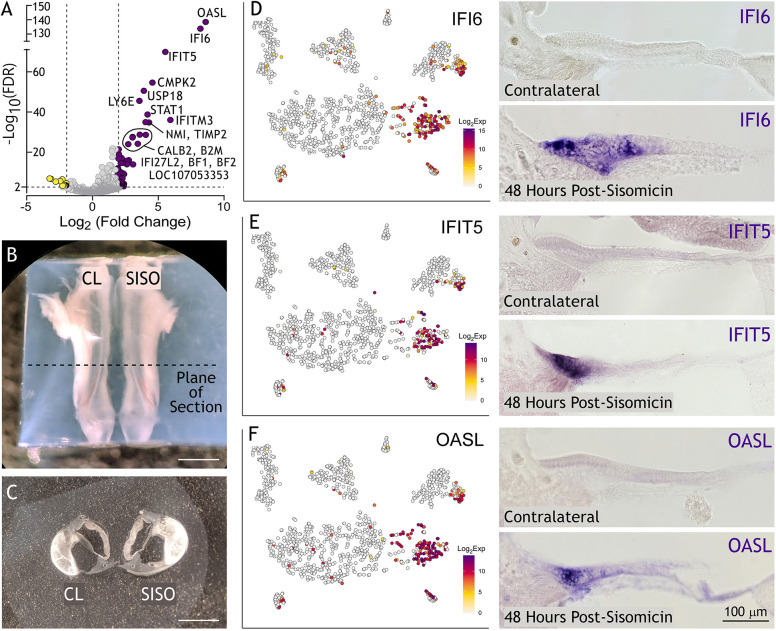
**Immune-related genes are upregulated in responding supporting cells.** (A) Volcano plot illustrating genes expressed in responding supporting cells (purple dots) at least fourfold higher and significantly different (FDR<0.01) compared with genes expressed in supporting cells (yellow dots). (B) Agarose-embedded basilar papilla from contralateral (CL) and sisomicin-infused (SISO) ears. The dotted line indicates the tonotopic middle of the basilar papilla from where vibratome sections are taken. (C) Resulting vibratome sections of CL and SISO basilar papillae. Ejected hair cells are visible in the SISO section. (D-F) tSNE plots (left panels) project log_2_ transformed, normalized expression counts for IFI6, IFIT5 and OASL. *In situ* hybridization (right panels) showing corresponding mRNA expression in P9 transverse sections, 48 h post-sisomicin damage. Hair cells are noticeably absent in the sisomicin-infused basilar papillae sections. Scale bars: 1 mm (B); 600 μm (C).

We hypothesized that single-cell RNA-seq would provide the resolution to distinguish whether immune-related genes are expressed by infiltrating immune cells or by sensory epithelial cells. The single cell analysis showed that the major responding cell group, signified by *IFI6* (Fig. 2I), also expressed the supporting cell marker *GSTT1L* (Fig. 2H). Thus, we inferred that supporting cells were the primary cell type showing upregulation of immune-related genes after damage. A small group of homogene cells, identified with the marker *LRP2*, also expressed *IFI6* (Fig. 2G,I). We conducted differential gene expression analysis on the responding supporting cell cluster (state S9 in Fig. 2B), comparing it with all other supporting cells (states S7 and S8 in Fig. 2B). This analysis revealed 43 upregulated genes (Fig. 3A and Table S4) using an FDR threshold of 0.01 and log_2_FC>2. We found minimal change of most supporting cell genes in the responding group, except for a modest reduction in *TECTA*, *SERPINF2*, *HEY2*, *FGFR3* and *TIMP3* (Table S4). Gene ontology analysis corroborated the bulk RNA-seq results, with the top upregulated terms falling into the category of interferon signaling response and modulation of an immune response (Fig. S4A).

Next, we validated the upregulated genes using colorimetric *in situ* hybridization. As this method is not inherently quantitative, we took appropriate measures for the side-by-side comparison of contralateral control versus the sisomicin-infused ear. We embedded the contralateral and sisomicin basilar papillae next to each other in agarose to ensure that transverse vibratome sections would come from equivalent tonotopic regions (Fig. 3B,C) and processed both sections together for *in situ* hybridization. *IFI6*, *IFIT5* and *OASL* were expressed robustly within the supporting cell layer of basilar papillae from sisomicin-infused ears, and predominantly on the medial/neural side of the epithelium (Fig. 3D-F). This suggests that the upregulated immune-related genes observed in the bulk RNA-seq data are being driven by the medial supporting cell population. To test whether the presence of immune cells contributed to the response, we immunolabeled basilar papilla sections for the macrophage marker TAP1 at 48 h post-sisomicin infusion (Fig. S4B). The supporting cell layer was actively proliferating at this time, as indicated by the incorporation of 5-ethynyl-2′-deoxyuridine (EdU), but was devoid of TAP1-positive cells (Fig. S4B). Infiltration of macrophages into the basilar papilla sensory epithelium after sisomicin-induced hair cell loss was not detectable. We noted a subcluster of responding homogene cells, entirely made up of cells from the 30- and 38-h time points (Fig. 2A,G; Fig. 2B – state S6; Table S3; Fig. S2). Differential expression of this responding group (state S6 in Fig. 2B) versus control cells (state S5 in Fig. 2B) revealed a similar collection of genes that we identified in responding supporting cells (Fig. S4C, Table S5). *In situ* hybridization confirmed expression of *IFI6* mRNA in homogene cells at 48 h post-sisomicin damage (Fig. S4D).

### Supporting cells use distinct regenerative mechanisms

**Fig. 4.**
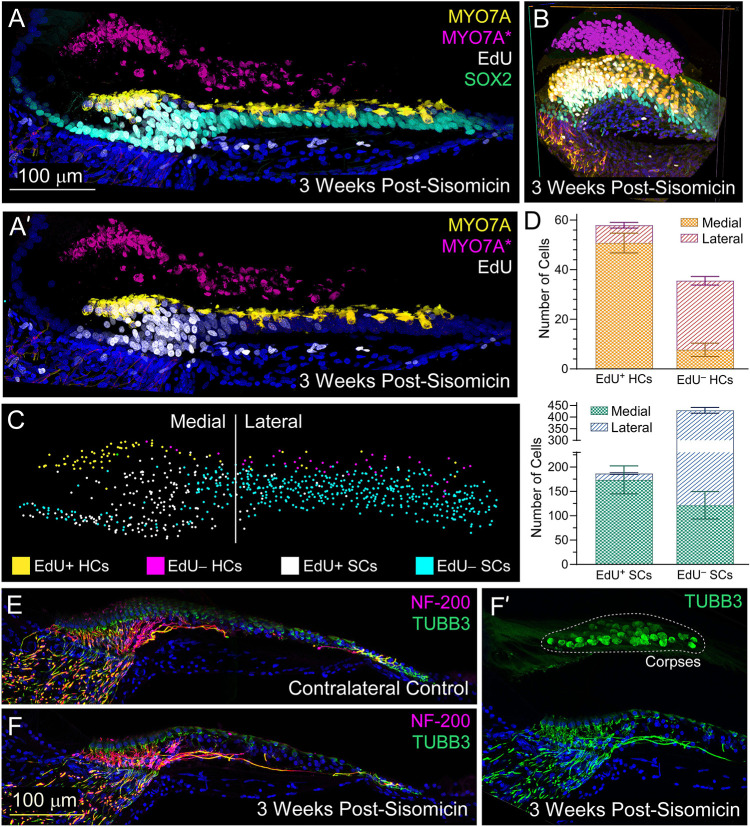
**Differential regenerative strategies revealed and quantitated at 3 weeks post-sisomicin.** Chickens were injected with EdU for 2 consecutive days, every 6 h, during the proliferative window, then sacrificed at 3 weeks. (A-B) Immunohistochemistry for MYO7A in yellow (new hair cells), SOX2 in cyan (supporting cells), EdU in white (cells that have proliferated) and DAPI in blue (cell nuclei) at 3 weeks post-sisomicin. MYO7A-positive hair cell corpses are false colored in magenta to distinguish them from newly regenerating hair cells in yellow. (A′) The SOX2 cyan channel is removed to clearly visualize EdU staining. (B) 3D Visualization-assisted analysis (Vaa3D) image of 3 weeks post-sisomicin basilar papilla. (C) The section pictured in A was manually quantitated in 3D virtual reality using syGlass software. (D) Cell counts from a total of 10 sections were quantitated as shown in C and are plotted in bar graphs. Data are mean±s.d. across the 10 sections. (E-F′) Immunohistochemistry for neurofilament-200 (NF-200) in magenta and β-tubulin III (TUBB3) in green, which both mark sensory ganglia, and DAPI in blue (cell nuclei). (E) Contralateral control. (F) Three weeks post-sisomicin. (F′) Same image as F but zoomed out to see hair cell corpses in the tectorial membrane, which confirms that these are damaged specimens.

Medial and lateral supporting cells employ different mechanisms to restore lost hair cells (Cafaro et al., 2007). We evaluated our damage/regeneration model at 3 weeks post-sisomicin with continual exposure to EdU to detect cells undergoing S-phase during the proliferative window of 30-80 h (Janesick et al., 2022). Virtually all regenerated hair cells on the medial/neural side of the epithelium displayed EdU-positive nuclei, leading us to infer that they emerged nearly exclusively from mitotic events (Fig. 4A-B). In contrast, the lateral/abneural side of the epithelium was regenerated mostly via phenotypic conversion, where a supporting cell morphs into a hair cell without dividing first (Fig. 4A-B). These results are comparable with previous observations conducted at 6 days post-damage (Cafaro et al., 2007). We quantified ten basilar papilla sections at 3 weeks post-sisomicin (Fig. 4C,D) using three-dimensional rendering in syGlass (Scientific Virtual Reality; https://www.syglass.io) and found that 87% of tall and 21% of short hair cells incorporated EdU (Fig. 4D). 59% of medial and 4% of lateral supporting cells incorporated EdU (Fig. 4D). We observed higher hair cell density on the medial/neural side of the epithelium at 3 weeks, compared with the more sparsely populated regenerated short hair cells, consistent with prioritization of mitotic tall hair cell restoration.

We attempted to subcluster the responding group of supporting cells (state S9 in Fig. 2B) further but could not distinguish medial and lateral cells based on markers identified by Janesick et al. (2021) (Fig. 2J). It should be noted that such clustering, even of control cells, is not robust because basilar papilla supporting cells are relatively homogeneous, in contrast to the distinct tall and short hair cell subtypes. Intriguingly, the *in situ* hybridizations in Fig. 3D-F revealed higher expression of immune-related genes on the medial side of the epithelium, coincident with EdU staining (Fig. 4A-B; Fig. S4B; Janesick et al., 2022).

### The JAK/STAT pathway is essential for upregulation of immune-related genes in supporting cells

**Fig. 5.**
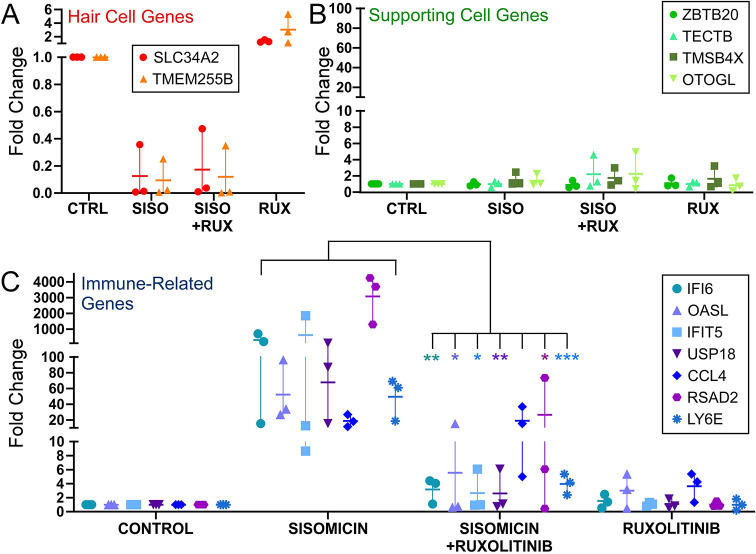
**JAK/STAT signaling is required for the immune-related response in culture.** Peeled sensory epithelia from P7 chickens were cultured according to Burns et al. (2008), with some modifications (described in the Materials and Methods). The epithelia were incubated overnight with or without sisomicin and with or without JAK/STAT inhibitor (RUX, ruxolitinib). The following day, sisomicin was removed and the epithelia were cultured 6 h with or without JAK/STAT inhibitors. Gene expression was determined by the 2^−ΔΔCT^ method using *HSPA8* as the reference gene. Data are reported as fold change over controls, representing three biological replicates. The vertical lines represent the data range, and the horizontal line is the mean of the replicates. (A) Hair cell genes, (B) supporting cell genes and (C) damage-response genes. Statistics were conducted using repeated-measures one-way ANOVA with Bonferroni's post-hoc analysis. All post-hoc comparisons are between sisomicin and sisomicin+ruxolitinib: *P≤0.05; **P≤0.01; ***P≤0.001. Modest upregulation of some HC and SC genes in response to RUX alone was observed in isolated experiments, but was neither consistent nor significant across all replicates and genes.

We next asked whether JAK/STAT signaling was required to upregulate the immune-related genes. Ruxolitinib (RUX) is a dual JAK1 and JAK2 small-molecule inhibitor (Quintás-Cardama et al., 2010). We tested both peeled and whole (organotypic) basilar papilla in culture to verify that immune-related genes are invoked by sisomicin, and found that both resulted in *IFI6* and *IFIT5* expression, but stronger inductions were observed with peeled epithelia. We cultured peeled sensory epithelium in the presence or absence of sisomicin with or without RUX overnight. Sisomicin was washed away, RUX was refreshed and the epithelium was incubated for 6 h with RUX only. mRNA was extracted and processed for qPCR to assess expression of selected immune-related and control genes. Sisomicin (with or without RUX) reduced the expression of hair cell markers *SLC34A2* and *TMEM255B*, which was expected as hair cells die in the presence of ototoxins (Fig. 5A). Expression of supporting cell markers *ZBTB20*, *TECTB*, *TMSB4X* and *OTOGL* persisted (Fig. 5B). Sisomicin-induced hair cell loss caused strong upregulation of mRNAs encoding *IFI6*, *IFIT5*, *OASL*, *USP18*, *CCL4*, *RSAD2* and *LY6E* (Fig. 5C). RUX blunted this response by 80-99% across all genes and three biological replicates with the exception of CCL4, which was unaffected by RUX (Fig. 5C). We concluded that the majority of immune-related genes induced after damage require JAK signaling.

### CALB2, USP18 and TRIM25 link responding supporting cells to new hair cells

**Fig. 6.**
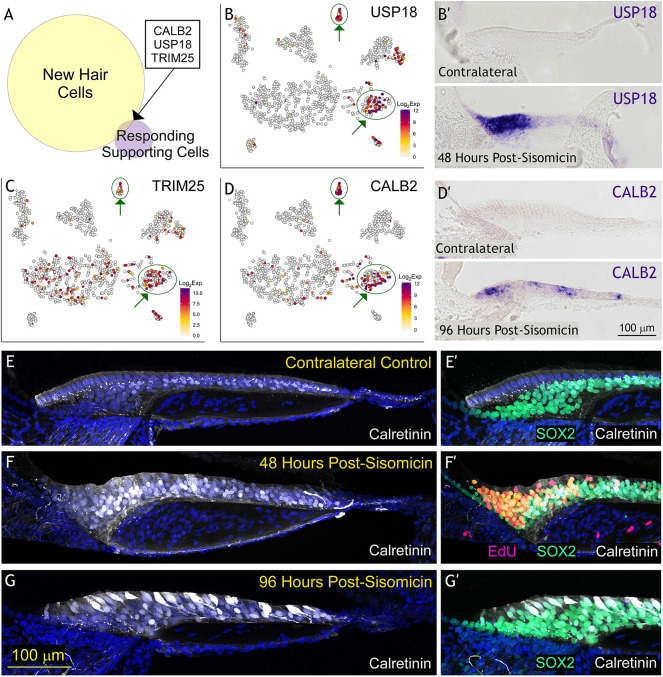
**An exclusive set of genes associates with both responding supporting cells and new hair cells.** (A) Venn diagram comparing statistically significant new hair cell markers and responding supporting cell markers. (B,D) tSNE plots project log_2_ transformed normalized expression counts for the only three genes (*USP18*, *TRIM25* and *CALB2*) that overlap between these two distinct clusters. (B′) *In situ* hybridization validates *USP18* mRNA expression in responding supporting cells in P9 transverse sections at 48 h post-sisomicin damage. (D′) *In situ* hybridization validates *CALB2* mRNA expression in new hair cells in P11 transverse sections at 96 h post-sisomicin damage. (E,F,G) Immunohistochemistry for CALB2 protein (also known as calretinin) is in white and DAPI is in blue at the timepoints listed. (E',F′,G′) Medial region of the same sections in E-G showing SOX2 (supporting cells) in green and calretinin in white. (F′) EdU was subcutaneously injected twice into chickens at ∼42 and 48 h post-sisomicin, and were sacrificed at 54 h post-sisomicin. Proliferative cells are labeled in magenta. EdU staining was not performed on control (E′) or 96 h (G′) specimens because we have never observed proliferation here (Janesick et al., 2022).

New hair cells are unequivocally detectable at 96 h post-sisomicin infusion in our single cell RNA-seq dataset (Fig. 2A,E; state S1 in Fig. 2B). These hair cells robustly express *ATOH1* (Fig. 2E), a crucial transcription factor in hair cell development (Bermingham et al., 1999), as well as other markers (Table S6). The responding supporting cells at 30 and 38 h (state S9 in Fig. 2B, Fig. 3) were distinct from the nascent regenerated hair cells at 96 h (state S1 in Fig. 2B). This gap exposed a limitation of our study where we would need to acquire additional cells from time points between 38 and 96 h to enable the building of a proper trajectory between responding supporting cells and new hair cells. In lieu of a trajectory analysis, we assessed the top-ranking genes (FDR<0.01 and log2FC>2) of the new hair cell and the responding supporting cell group, and found three genes present in both: *CALB2*, *USP18* and *TRIM25* (Fig. 6A-D). These genes link the responding supporting cell population with the newly regenerated nascent hair cells, and we hypothesize that they represent ‘cornerstones’ of a trajectory of presumed gene expression changes towards new hair cells. We confirmed *USP18* and *CALB2* expression by *in situ* hybridization at 48 and 96 h post-sisomicin, respectively (Fig. 6B′,D′). In mammals, *USP18* competes with JAK, thus preventing phosphorylation of downstream substrates, including STATs (Malakhova et al., 2006; Wilmes et al., 2015). We hypothesized that *USP18* acts by suppressing JAK/STAT response genes in newly regenerated hair cells.

We explored *CALB2* in greater depth because it is also expressed in both responding supporting cells and new hair cells, and antibodies to the protein are available (Dechesne et al., 1994; Edmonds et al., 2000; Ellwanger et al., 2018). We evaluated CALB2 protein expression in regenerating and control sensory epithelia (Fig. 6E-G′). In contralateral controls, CALB2 was present mainly in synaptic terminals appearing as punctate staining, and weakly in supporting cell nuclei (Fig. 6E,E′). At 48 h post-sisomicin infusion, CALB2 was found in both medial and lateral supporting cells (CALB2/SOX2^+^), with a strong signal in the nuclei (Fig. 6F,F′). EdU staining in the same specimen confirmed that these supporting cells were in the proliferative phase of regeneration (Fig. 6F′). At 96 h post-sisomicin, CALB2 was found in supporting cells largely in the nuclei and in new hair cells where the protein is cytoplasmic and nuclear (Fig. 6G,G′). Moreover, we detected CALB2 expression as early as embryonic day 9 in the chicken basilar papilla (Fig. S5B), prior to the onset of OTOF expression in hair cells at E11 (Fig. S5C,D). It was previously known that CALB2 is an important marker of differentiated hair cells with functional physiological roles as a calcium sensor (reviewed by Camp and Wijesinghe, 2009). Here, we show that CALB2 can also be detected in proliferating medial supporting cells in the chicken basilar papilla after damage, and that the protein is abundant in regenerated nascent hair cells. *CALB2*, therefore, functions as both a developmental and mature hair cell gene in different contexts, in addition to distinguishing mammalian sensory ganglia neuronal subtypes (Shrestha et al., 2018; Sun et al., 2018).

## DISCUSSION

### Hair cell loss induces immune-related gene expression in basilar papilla supporting cells

How non-mammalian species restore hearing after damage is among the most inspirational unanswered questions in the regeneration field. To address this question, we performed an RNA-seq analysis of the regenerating chicken basilar papilla. We collected cells at 48 h post-damage for bulk RNA-seq and at 30, 38 and 96 h post-damage for single-cell RNA-seq. Our results revealed a striking induction of immune-related genes in supporting cells 30, 38, and 48 h after sisomicin damage.

Inflammation and oxidative stress responses to ototoxic insult have been reported (reviewed by Dinh et al., 2015), and infiltrating leukocytes are often implicated in these responses (Bhave et al., 1998; Hirose et al., 2017; Kaur et al., 2015; Ladrech et al., 2007; O'Halloran and Oesterle, 2004; Tornabene et al., 2006; Warchol, 1997; Warchol et al., 2012). Other observations indicate that supporting cells emulate immune cells and are ‘glial-like’, expressing GFAP and GLAST (Hayashi et al., 2020; Matsunaga et al., 2020; Monzack and Cunningham, 2013; Wan et al., 2013). Our baseline analysis of the chicken basilar papilla demonstrated strong expression of the astrocytic marker *ZBTB20* in supporting cells (Janesick et al., 2021; Nagao et al., 2016). Therefore, supporting cells might be suited for neuroimmune responses.

Previous reports investigating gene expression changes following hair cell damage in the chicken inner ear did not include single-cell analysis and were consequently unable to link immune-response genes to specific cell types (Table S1). Our data suggest that damage induces strong upregulation of immune-related genes in supporting cells without significant contribution from blood cell infiltration. This observation is supported by a recent study using cultured streptomycin-treated chicken basilar papillae (Matsunaga et al., 2020). Interferon receptors (Goossens et al., 2013) are not expressed in the basilar or utricular sensory epithelium in our single-cell datasets (Janesick et al., 2021; Scheibinger et al., 2021 preprint). We also found no evidence for expression of interferon (e.g. IFNα, IFNβ and IFNγ) genes in basilar papilla sensory epithelium post-sisomicin, but we cannot exclude the possibility that potent ligands are expressed in surrounding tissue not included in our single-cell analysis. We compared known receptors used by avian species to invoke an interferon response (Neerukonda and Katneni, 2020) with our single-cell datasets but did not find any strong leads. The RNA-sensors, TLR3 and OASL were induced after damage and are expressed in supporting cells at homeostasis, which could point to alternative potential activation mechanisms. Regardless of the initial trigger, immune-related genes are often regulated by JAK/STAT signaling, although other non-canonical pathways (MAPK, mTOR and pI3-kinase) can contribute to the response. We found that JAK/STAT signaling was required because Ruxolitinib effectively blocked expression of the damage-induced immune-related genes, as also shown by Matsunaga et al. (2020). We cannot exclude the possibility that an initial signaling event could occur via a non-JAK/STAT mechanism, which then subsequently promotes expression of interferon-response genes, leading to JAK/STAT activation later.

Like all epithelial cells, supporting cells function as a barrier to separate distinct environments. After hair cell death, supporting cells expand their apical surfaces to compensate for lost hair cells and to preserve epithelial integrity (reviewed by Francis and Cunningham, 2017). In other epithelial systems, there is ample evidence that cells not from the hematopoietic lineage can provide a barrier to the external environment and mimic some of the actions of immune cells. For example, epidermal keratinocytes and dermal fibroblasts can secrete cytokines, apolipoproteins and antimicrobial peptides (reviewed by Nguyen and Soulika, 2019). Zebrafish ectoderm-derived ‘metaphocytes’ share transcriptomic and morphological characteristics with macrophages (Lin et al., 2019). These metaphocytes sample soluble antigens directly from the external environment and transfer them to macrophages, but their developmental lineage is different from the macrophages they service (epidermis versus mesodermal germ layer).

Whether the upregulation of the immune-related genes that we observe is a trigger for regeneration or a mechanism to mitigate the detrimental effects of dying cells requires future study. In other systems, cytokine signaling is linked to epithelial repair and also to cell turnover and proliferation. After a colonic injury, the intestinal epithelium mounts an interferon response that is required for regeneration and depends on EGFR and the ligand AREG (McElrath et al., 2021). Mice lacking the interferon receptors show impaired epithelial proliferation, compromising the mucosal barrier (McElrath et al., 2021). Chronic viral infection also promotes epithelial proliferation in the kidney, liver and salivary glands via type I interferon signaling (Sun et al., 2015). In the *Drosophila* midgut, an injury-induced cytokine response promotes stem cell and enterocyte division and differentiation, respectively (Jiang et al., 2009). Therefore, there is support across multiple models for cytokine responses leading to S-phase entry and regeneration.

### Controlling immune-related gene expression in the chicken basilar papilla

As H. W. Longfellow wrote, ‘Great is the art of beginning, but greater is the art of ending’ (Longfellow, 1882). Signal inactivation mechanisms are built into developmental and regenerative systems to prevent runaway feed-forward signaling. The chicken basilar papilla appears to have excellent control over the transient upregulation of immune-related genes. Despite the huge increase in *IFI6*, *IFIT5* and *OASL* (among other genes) expression at 48 h post-sisomicin, their expression is almost non-existent in regenerated nascent hair cells. We suggest that *USP18* plays a key role in shutting down JAK/STAT response genes, considering its known role in terminating the interferon signaling cascade (Basters et al., 2018; Hou et al., 2021). *USP18* is one of few genes that is expressed in both supporting cells at 30 and 38 h (FC=15, FDR=3.1×10^−51^), as well as nascent hair cells at 96 h post-sisomicin (FC=60, FDR=9.0×10^−21^). Another negative regulator of JAK/STAT signaling, *SOCS3*, is expressed by new hair cells (FC=6.3, FDR=1.7×10^−11^), and is also implicated in hair cell regeneration in zebrafish (Liang et al., 2012).

*TRIM25* is another gene that straddles responding supporting cells (FC=5.1, FDR=3.8×10^−10^) and new hair cells (FC=12.6, FDR=2.4×10^−14^). *TRIM25* is a E3 ubiquitin ligase that participates in the innate immune response, regulation of cell proliferation and cancer cell invasion (reviewed by Martín-Vicente et al., 2017). TRIM25 is a co-factor in LIN28-mediated uridylation of pre-let-7 (Choudhury et al., 2014). Intriguingly, LIN28B is expressed in the mammalian cochlea and controls the ability of neonatal murine auditory supporting cells to generate hair cells through mTOR signaling (Li and Doetzlhofer, 2020). Neither *LIN28A* nor *LIN28B* are expressed in the chicken basilar papilla by single-cell RNA-seq. Hence, this neonatal mammalian mechanism to generate hair cells is either not conserved or uses different gene sets in avian species. TRIM25 positively regulates inflammatory cytokine signaling through K63-linked ubiquitylation of RIG-I, but also negatively regulates cytokines via stabilization of FAT10, and the ubiquitylation of MAVS (reviewed by Martín-Vicente et al., 2017). The RIG-I homolog is absent in chickens (Santhakumar et al., 2017), thus likely altering TRIM25 functionality in comparison with mammals, in the context of positively or negatively regulating cytokine responses.

### CALB2 is expressed in supporting cells after damage and in newly regenerated hair cells

*CALB2* belongs to the small group of genes expressed in both responding supporting cells (FC=12, FDR=1.9×10^−29^) and new hair cells (FC=745, FDR=2.1×10^−68^). *CALB2* encodes calretinin, a calcium sensor expressed in distinct neuronal populations innervating sensory organs, including retinal ganglion cells, the granular layer of the cerebellar cortex and brainstem auditory neurons (reviewed by Camp and Wijesinghe, 2009). Calretinin modulates neuronal excitability as a slow calcium chelator at resting intracellular Ca^2+^ levels and a fast-onset buffer at elevated intracellular Ca^2+^ levels (Gall et al., 2003; Schwaller, 2009). In the organ of Corti, calretinin is initially expressed in inner and outer hair cells but disappears in outer hair cells upon maturity, coinciding with the loss of afferents (Dechesne et al., 1994). In the chicken basilar papilla, we found punctate calretinin expression, presumably in nerve terminals associated with tall hair cells, and observed crescent-shaped staining around short hair cells. We also detected calretinin in superior tall hair cells and faintly in the nuclei of supporting cells.

Forty-eight hours after damage, nuclear expression of calretinin in supporting cells intensified and persisted as newly regenerated calretinin-positive hair cells emerged. To our knowledge, this is the first time calretinin was observed strongly in proliferating supporting cells during development or regeneration. In the rat utricle, calretinin was observed only in post-mitotic, differentiating hair cells (Zheng and Gao, 1997). In the chicken utricle, calretinin was detected in new hair cells of asymmetric pairs but not in the supporting cell layer during both natural turnover and regeneration (Stone and Rubel, 1999). Therefore, the supporting cell expression of calretinin is unique to the regenerating chicken basilar papilla. The nuclear staining we observed for calretinin is also unusual, although there is precedent for nuclear labeling in turtle hair cells (Hackney et al., 2003), as well as nuclear translocation of calretinin in human colon carcinoma cells in response to vitamin D (Schwaller and Herrmann, 1997). The vitamin D receptor is not expressed in the chicken basilar papilla sensory epithelium; therefore, it is unlikely that calretinin subcellular localization is regulated via this process. Whether hair cell differentiation requires early, nuclear CALB2 localization is unknown. In the mammalian inner ear, calretinin expression in new hair cells requires ATOH1 (Bermingham et al., 1999). It is unlikely that ATOH1 would require calretinin expression during hair cell development, despite our observation of an abundance of CALB2 mRNA and calretinin protein before new hair cells emergence and before *ATOH1* expression. Rather, we surmise that calretinin expression could reflect unique calcium needs of damaged supporting cells.

### Regenerative strategies inferred at 3 weeks post-sisomicin damage

There are two modes of hair cell regeneration in the chicken sensory epithelium: proliferation and phenotypic conversion. Hair cells on the medial/neural side of the epithelium incorporate thymidine analogs after damage. In contrast, hair cells on the lateral/abneural side transdifferentiate from a supporting cell to a hair cell directly, rather than undergoing a mitotic event (reviewed by Janesick and Heller, 2019). Previous studies focused on the chicken basilar papilla 6 days post-gentamicin and found that 34% of newly generated short hair cells incorporated bromodeoxyuridine, compared with 81% of new tall hair cells (Cafaro et al., 2007). Because the time course of regeneration is in the order of months (Duckert and Rubel, 1993; Girod et al., 1991; Tucci and Rubel, 1990), these studies represent an early snapshot before the full complement of hair cells are restored. We extended our analysis to 3 weeks post-sisomicin and confirmed that hair cells on the medial/neural side of the epithelium were regenerated predominantly by mitotic events (87%). Only 21% of new lateral hair cells incorporated EdU.

In the vestibular system, supporting cell replenishment was postulated as a mechanism to replace supporting cells that had directly converted to hair cells without dividing (Scheibinger et al., 2018). However, we found that only 4% of lateral basilar papilla supporting cells proliferated. For example, across all sections of individual basilar papillae, we quantified on average 28 EdU-negative short hair cells but only 13 EdU-positive supporting cells beneath them. We envision alternative conclusions from this result. The first is that replenishment of supporting cells was simply incomplete or not required. The ratio of lateral supporting cells to short hair cells is over twice the ratio of medial supporting cells to tall hair cells (Goodyear and Richardson, 1997; Janesick and Heller, 2019), suggesting that an abundant reservoir of supporting cells is available. It would be informative to investigate whether the lateral supporting cells could eventually be depleted after repeated rounds of damage and regeneration. The second conclusion is that our window of EdU administration was too early/short and might not have detected a second/later wave of supporting cell division. A third conclusion is that medial supporting cells could migrate to the lateral side over time, but the time course is too gradual/slow to observe. Indeed, we found that 3.4 times more medial supporting cells are EdU positive compared with tall hair cells, suggesting that supporting cells are not simply replacing themselves with asymmetric divisions, but might also undergo symmetric divisions, which we speculate could replenish supporting cells that migrated to the lateral side.

The possibility that regeneration of the sensory epithelium is incomplete (both at the hair cell and supporting cell level) has merit because previous reports have observed that functional hearing is not entirely restored. In pigeons and chickens, aminoglycoside damage triggers a regenerative response that eventually yields substantial hearing recovery. However, high-frequency regions (>1.5 kH) still exhibit a 20-30 decibel permanent threshold shift at 4-5 months post-damage (reviewed by Dooling et al., 2008; Saunders and Salvi, 2008). We observed that the timescale of short hair cell replenishment is slower than for tall hair cells. The density of tall hair cells at 3 weeks approximates control levels, whereas the short hair cell regions are more sparsely populated. If tall hair cells fulfill a similar role to mammalian inner hair cells, then it would make sense to prioritize tall hair cell regeneration to restore basic hearing function.

### Conclusions

The present study contributes mechanistic knowledge about avian hair cell regeneration. Single cell RNA-seq and *in situ* validation revealed that supporting cells, the facultative stem cells of the avian inner ear, robustly upregulate immune-related genes, which is mediated by JAK/STAT signaling. No distinct candidate receptor and ligand combination emerged from our analysis, exposing the need for further studies. Our dataset provides a rich resource for such studies and motivates the investigation of JAK/STAT signaling in the damaged mammalian inner ear. This signaling pathway is highly potent, leading to inflammation, cytokine storms and fibrosis. We provided evidence that, in the regenerating basilar papilla, expression of immune-related genes is tightly controlled, such that 4 days after damage, they are no longer expressed in newly regenerated hair cells. Instead, the newly regenerated hair cells display unique combinations of genes that differ from naturally generated hair cells, which unveils differences between development/homeostasis and regeneration. Our collection of gene expression changes during avian hair cell regeneration provides a valuable resource with respect to devising strategies for hair cell regeneration in mammals.

## MATERIALS AND METHODS

### Bulk (population-based) RNA-sequencing and analysis

All surgical procedures and dissections were performed according to Janesick et al. (2021, 2022) and were approved by the Stanford University Institutional Animal Care and Use Committee. Each epithelium was placed into 100 µl RNAqueous Lysis Solution in a nuclease-free tube, quickly triturated and vortexed, then frozen at −80°C. RNA was extracted using the RNAqueous-Micro Total RNA Isolation Kit, following the manufacturer's instructions. Total RNA was assessed with the Agilent 2100 Bioanalyzer at the Stanford Functional Genomics Facility (SFGF). The RNA yield from one peeled, sensory epithelium is ∼20-30 ng with RIN values ranging from 8.2 to 9.5. Library preparation was performed by SFGF using the SMART-Seq v4 Ultra Low Input RNA Kit (Takara Bio). The libraries were sequenced on the Illumina HiSeq 4000 platform (2×75 bp paired-end reads). *n*=3 for control and for sisomicin, where *n* represents one cold peeled epithelium. No pooling of samples was carried out, thus minimizing the averaging of biological variability.

Data from the SFGF were provided as raw Fastq files which we submitted to BasePair (www.basepairtech.com). Reads were pre-processed with fastp v0.19.4 (Chen et al., 2018) and aligned with STAR v2.6 (Dobin et al., 2013) to *Gallus gallus* genome GRCg6a. BasePair provided gene feature counts (see Table S7), which we processed by following and combining the methods outlined in two software tool articles (Chen et al., 2016; http://www.nathalievialaneix.eu/doc/html/solution-edgeR-rnaseq.html). We filtered out 10,806 of 22,758 genes by employing the HTSFilter v3.8 (Rau et al., 2013) on trimmed-mean-of-M-values (TMM) normalized count data to yield a statistically robust, non-arbitrary threshold for low abundance and constant genes. We imported filtered genes into edgeR v2.14 for differential gene expression analysis (Robinson et al., 2010). Mean-difference volcano and per-sample expression plots were generated using Glimma (Su et al., 2017). The volcano plot in Fig. 1 was manually colored using Canvas X Draw v20. A heatmap of log_2_(counts-per-million) values was created using Pheatmap v1.0.12. Gene ontology analysis was conducted using PathfindR v1.6.1 with the Reactome database (Jassal et al., 2019; Ulgen et al., 2019).

### Single cell RNA-sequencing and analysis

All methods for single cell RNA-seq and data processing were conducted as described by Janesick et al. (2021). The experimental design is outlined in Fig. S2. Cold-peeled epithelia from four to six animals belonging to one clutch of chickens were dissociated, washed and submitted for FACS. Cells were index sorted into two 96-well plates (batches 1 and 2), after excluding for debris, doublets and low viability. This process was repeated twice more (per timepoint) with different clutches of chickens (batches 3 and 4, and batches 5 and 6). Cells were submitted to the Stanford Functional Genomics Facility where they were processed using the Smart-Seq2 protocol (Picelli et al., 2014). cDNA size distribution was assessed for each individual cell using an Agilent 2100 Bioanalyzer and 364 cells were selected for library construction. All cell libraries were sequenced together with an Illumina NextSeq 500 sequencer aiming for 400 million 150 bp, paired end reads resulting in about 1 million reads per cell.

Barcode demultiplexing and read mapping against the *Gallus gallus* genome (release GRCg6a; https://www.ncbi.nlm.nih.gov/assembly/GCF_000002315.6) was conducted as described by Janesick et al. (2021). Quality control was conducted in R using scater v1.20.1 (McCarthy et al., 2017). Information-poor cells were removed based on the following thresholds: number of raw counts >10,000; number of expressed genes >100. Lowly expressed genes were also removed (average counts >0.1). Normalization was performed with the SCnorm package (Bacher et al., 2017). Non-linear dimensionality reduction and cell clustering were conducted using the CellTrails package (Ellwanger et al., 2018). Differential expression analysis between clusters was performed with EdgeR (Robinson et al., 2010) on log_2_ normalized expression counts. False discovery rates are reported as Benjamini and Hochberg-corrected *P*-values. tSNE and volcano plots were generated as previously described (Janesick et al., 2021). Because blood cells are easily identified bioinformatically, we chose to include them in the tSNE plots. Violin plots were generated in ggplot2 v3.3.4. The Venn diagram in Fig. 6A was generated by comparing differentially expressed genes in Tables S4 and S6 using the FDR cut-off of 0.01 and log_2_FC>2.

### Vibratome sectioning, *in situ* hybridization and immunohistochemistry

Basilar papillae ducts in bone were processed for vibratome sectioning and *in situ* hybridization or immunohistochemistry as previously described (Janesick et al., 2021; Scheibinger et al., 2022). T7 adapted RNA probe templates were prepared via PCR amplification with primers listed in Table S8. 5-ethynyl-2′-deoxyuridine (EdU) staining was conducted as previously described (Janesick et al., 2022). Antibody sources for immunohistochemistry are provided in Table S9.

### Three weeks post-sisomicin experiment

Maintaining animals for longer than 1 week post-sisomicin requires extra attention to animal welfare due to vestibular defects from the sisomicin infusion (Janesick et al., 2022). Furthermore, quantitation of EdU-positive cells must be conducted with a constant influx of EdU into the inner ear. Otherwise, an EdU-negative, MYO7A-positive new hair cell could be misconstrued for a phenotypically converted supporting cell, when, instead, the cell simply did not incorporate EdU due to lack of EdU bioavailability. We determined that subcutaneously injected EdU will be available to the inner ear for ∼6 h (Janesick et al., 2022). Some researchers choose a mini-osmotic for the constant delivery of thymidine analogs (Cafaro et al., 2007). We chose to re-administer 50 mg/kg EdU in 200 µl PBS/DMSO subcutaneously every 6 h. We started dosing EdU at 30 h post-sisomicin and continued every 6 h until the last dose at 80 h (over 72 h post-sisomicin). This dosing regimen ensured that we would cover the proliferative window (Janesick et al., 2022).

We quantitated hair cells and supporting cells using a 3D virtual reality strategy outlined by Janesick et al. (2022). Briefly, transverse vibratome sections were subjected to EdU detection using the Click-iT EdU Cell Proliferation Kit (ThermoFisher), then immunostained with SOX2, MYO7A and DAPI (Scheibinger et al., 2022). The sections were cleared and then imaged at 1.0× zoom with a confocal microscope at 40× magnification (Zeiss LSM880; Plan-Apochromat 1.3 numerical aperture, oil immersion) using Zen Black acquisition software at a voxel size of 0.208×0.208×0.371 µm and z-depth of 80 µm. The image data were imported into syGlass software (Pidhorskyi et al., 2018 preprint), which interfaces with SteamVR tracking and the Oculus Rift virtual reality headset, touch controllers and constellation sensors. Using the ‘count’ function, individual cells were manually annotated in 3D virtual reality. The *x*, *y* and *z* coordinates of each count was exported into Vaa3D (Fig. 4B), GraphPad Prism v9 (for bar chart in Fig. 4C) and MATLAB vR2017b (for 3D graphing using the scatter3 function – Fig. 4D).

### Epithelial cell cultures and quantitative RT-PCR

Culturing of sensory epithelia was conducted as previously described for the utricle (Burns et al., 2008), with some adaptations. 14* *mm MatTek dishes were coated with CellTak according to the manufacturer's instructions, allowing 20 min for absorption, and washing afterwards with sterile water. Peeled sensory epithelia in Medium 199+10 µg/ml ciprofloxacin (no serum) were mouth pipetted onto the MatTek dish. The microwell was then filled with the same culture medium. The basilar papilla epithelia were arranged with an eyebrow such that the hair bundles were facing upwards. A circular 12* *mm coverglass was placed over the microwell of each dish. The dish was centrifuged at 400 ***g*** for 10 min in an Eppendorf 5810R centrifuge to promote adhesion of the sensory epithelia. After centrifugation, the entire dish was slowly filled with Medium 199+cipro (no serum), the circular coverglass was carefully removed and the sensory epithelia were incubated for 1 h at 39°C in 5% CO_2_. This medium was then replaced with sisomicin (0.1 mg/ml), with sisomicin+JAK/STAT inhibitor (500 nM) or with control vehicle in Medium 199+cipro+10% FBS, and incubated overnight at 39°C in 5% CO_2_. The following day, the medium was replaced with sisomicin-free medium, but with the continuation of inhibitors for another day.

Total RNA was extracted using the RNAqueous-Micro Kit (ThermoFisher) and reverse transcribed into cDNA using SuperScript IV (Invitrogen). QPCR was performed using Bio-Rad CFX96 Touch Real-Time PCR machine with primer sets listed in Table S10 and SYBR green detection. Each primer set amplified a single band as determined by gel electrophoresis and melting curve analysis. QPCR data were analyzed using the 2^−ΔΔCt^ method (Livak and Schmittgen, 2001) relative to HSPA8 (housekeeping gene). Statistics were conducted in GraphPad Prism version 9.1.2.

## Supplementary Material

10.1242/develop.200113_sup1 Supplementary information

## References

[DEV200113C1] Bacher, R., Chu, L.-F., Leng, N., Gasch, A. P., Thomson, J. A., Stewart, R. M., Newton, M. and Kendziorski, C. (2017). SCnorm: robust normalization of single-cell RNA-seq data. *Nat. Methods* 14, 584-586. 10.1038/nmeth.4263 28418000 PMC5473255

[DEV200113C2] Basters, A., Knobeloch, K. and Fritz, G. (2018). How USP18 deals with ISG15-modified proteins: structural basis for the specificity of the protease. *FEBS J.* 285, 1024-1029. 10.1111/febs.14260 28881486

[DEV200113C3] Benkafadar, N., Janesick, A., Scheibinger, M., Ling, A. H., Jan, T. A. and Heller, S. (2021). Transcriptomic characterization of dying hair cells in the avian cochlea. *Cell Rep.* 34, 108902. 10.1016/j.celrep.2021.108902 33761357

[DEV200113C4] Bermingham-McDonogh, O. and Rubel, E. W. (2003). Hair cell regeneration: winging our way towards a sound future. *Curr. Opin. Neurobiol.* 13, 119-126. 10.1016/S0959-4388(03)00018-7 12593990

[DEV200113C5] Bermingham, N. A., Hassan, B. A., Price, S. D., Vollrath, M. A., Ben-Arie, N., Eatock, R. A., Bellen, H. J., Lysakowski, A. and Zoghbi, H. Y. (1999). Math1: an essential gene for the generation of inner ear hair cells. *Science* 284, 1837. 10.1126/science.284.5421.1837 10364557

[DEV200113C6] Bhave, S. A., Oesterle, E. C. and Coltrera, M. D. (1998). Macrophage and microglia-like cells in the avian inner ear. *J. Comp. Neurol.* 398, 241-256. 10.1002/(SICI)1096-9861(19980824)398:2<241::AID-CNE6>3.0.CO;2-0 9700569

[DEV200113C7] Brockes, J. P. and Kumar, A. (2008). Comparative aspects of animal regeneration. *Annu. Rev. Cell Dev. Biol.* 24, 525-549. 10.1146/annurev.cellbio.24.110707.175336 18598212

[DEV200113C8] Burns, J., Christophel, J. J., Collado, M. S., Magnus, C., Carfrae, M. and Corwin, J. T. (2008). Reinforcement of cell junctions correlates with the absence of hair cell regeneration in mammals and its occurrence in birds. *J. Comp. Neurol.* 511, 396-414. 10.1002/cne.21849 18803241 PMC2582022

[DEV200113C9] Cafaro, J., Lee, G. S. and Stone, J. S. (2007). Atoh1 expression defines activated progenitors and differentiating hair cells during avian hair cell regeneration. *Dev. Dyn.* 236, 156-170. 10.1002/dvdy.21023 17096404

[DEV200113C10] Camp, A. J. and Wijesinghe, R. (2009). Calretinin: modulator of neuronal excitability. *Int. J. Biochem. Cell Biol.* 41, 2118-2121. 10.1016/j.biocel.2009.05.007 19450707

[DEV200113C11] Chen, Y., Lun, A. T. L. and Smyth, G. K. (2016). From reads to genes to pathways: differential expression analysis of RNA-Seq experiments using Rsubread and the edgeR quasi-likelihood pipeline. *F1000Research* 5, 1438. 10.12688/f1000research.8987.1 27508061 PMC4934518

[DEV200113C12] Chen, S., Zhou, Y., Chen, Y. and Gu, J. (2018). fastp: an ultra-fast all-in-one FASTQ preprocessor. *Bioinformatics* 34, i884-i890. 10.1093/bioinformatics/bty560 30423086 PMC6129281

[DEV200113C13] Choudhury, N. R., Nowak, J. S., Zuo, J., Rappsilber, J., Spoel, S. H. and Michlewski, G. (2014). Trim25 is an RNA-specific activator of Lin28a/TuT4-mediated uridylation. *Cell Rep.* 9, 1265-1272. 10.1016/j.celrep.2014.10.017 25457611 PMC4542301

[DEV200113C14] Dechesne, C. J., Rabejac, D. and Desmadryl, G. (1994). Development of calretinin immunoreactivity in the mouse inner ear. *J. Comp. Neurol.* 346, 517-529. 10.1002/cne.903460405 7983242

[DEV200113C15] Dinh, C. T., Goncalves, S., Bas, E., Van De Water, T. R. and Zine, A. (2015). Molecular regulation of auditory hair cell death and approaches to protect sensory receptor cells and/or stimulate repair following acoustic trauma. *Front. Cell. Neurosci.* 9, 96. 10.3389/fncel.2015.00096 25873860 PMC4379916

[DEV200113C16] Dobin, A., Davis, C. A., Schlesinger, F., Drenkow, J., Zaleski, C., Jha, S., Batut, P., Chaisson, M. and Gingeras, T. R. (2013). STAR: ultrafast universal RNA-seq aligner. *Bioinformatics* 29, 15-21. 10.1093/bioinformatics/bts635 23104886 PMC3530905

[DEV200113C17] Dooling, R. J., Dent, M. L., Lauer, A. M. and Ryals, B. M. (2008). Functional recovery after hair cell regeneration in birds. In *Hair Cell Regeneration, Repair, and Protection* (ed. R. J. Salvi, A. N. Popper and R. R. Fay), pp. 117-140. New York, NY: Springer New York.

[DEV200113C18] Duckert, L. G. and Rubel, E. W. (1993). Morphological correlates of functional recovery in the chicken inner ear after gentamycin treatment. *J. Comp. Neurol.* 331, 75-96. 10.1002/cne.903310105 8320349

[DEV200113C19] Edmonds, B., Reyes, R., Schwaller, B. and Roberts, W. M. (2000). Calretinin modifies presynaptic calcium signaling in frog saccular hair cells. *Nat. Neurosci.* 3, 786-790. 10.1038/77687 10903571

[DEV200113C20] Ellwanger, D. C., Scheibinger, M., Dumont, R. A., Barr-Gillespie, P. G. and Heller, S. (2018). Transcriptional dynamics of hair-bundle morphogenesis revealed with cell trails. *Cell Rep.* 23, 2901-2914.e13. 10.1016/j.celrep.2018.05.002 29874578 PMC6089258

[DEV200113C21] Francis, S. P. and Cunningham, L. L. (2017). Non-autonomous cellular responses to ototoxic drug-induced stress and death. *Front. Cell. Neurosci.* 11, 252. 10.3389/fncel.2017.00252 28878625 PMC5572385

[DEV200113C22] Gall, D., Roussel, C., Susa, I., D'Angelo, E., Rossi, P., Bearzatto, B., Galas, M. C., Blum, D., Schurmans, S. and Schiffmann, S. N. (2003). Altered neuronal excitability in cerebellar granule cells of mice lacking calretinin. *J. Neurosci.* 23, 9320-9327. 10.1523/JNEUROSCI.23-28-09320.2003 14561859 PMC6740583

[DEV200113C23] Girod, D. A., Tucci, D. L. and Rubel, E. W. (1991). Anatomical correlates of functional recovery in the avian inner ear following aminoglycoside ototoxicity. *Laryngoscope* 101, 1139-1149. 10.1288/00005537-199111000-00001 1943414

[DEV200113C24] Goodyear, R. and Richardson, G. (1997). Pattern formation in the basilar papilla: evidence for cell rearrangement. *J. Neurosci.* 17, 6289-6301. 10.1523/JNEUROSCI.17-16-06289.1997 9236239 PMC6568370

[DEV200113C25] Goossens, K. E., Ward, A. C., Lowenthal, J. W. and Bean, A. G. D. (2013). Chicken interferons, their receptors and interferon-stimulated genes. *Dev. Comp. Immunol.* 41, 370-376. 10.1016/j.dci.2013.05.020 23751330

[DEV200113C26] Hackney, C. M., Mahendrasingam, S., Jones, E. M. C. and Fettiplace, R. (2003). The distribution of calcium buffering proteins in the turtle cochlea. *J. Neurosci.* 23, 4577-4589. 10.1523/JNEUROSCI.23-11-04577.2003 12805298 PMC6740801

[DEV200113C27] Hayashi, Y., Suzuki, H., Nakajima, W., Uehara, I., Tanimura, A., Himeda, T., Koike, S., Katsuno, T., Kitajiri, S., Koyanagi, N. et al. (2020). Cochlear supporting cells function as macrophage-like cells and protect audiosensory receptor hair cells from pathogens. *Sci. Rep.* 10, 6740. 10.1038/s41598-020-63654-9 32317718 PMC7174420

[DEV200113C28] Hirose, K., Rutherford, M. A. and Warchol, M. E. (2017). Two cell populations participate in clearance of damaged hair cells from the sensory epithelia of the inner ear. *Hear. Res.* 352, 70-81. 10.1016/j.heares.2017.04.006 28526177 PMC5544544

[DEV200113C29] Hou, J., Han, L., Zhao, Z., Liu, H., Zhang, L., Ma, C., Yi, F., Liu, B., Zheng, Y. and Gao, C. (2021). USP18 positively regulates innate antiviral immunity by promoting K63-linked polyubiquitination of MAVS. *Nat. Commun.* 12, 2970. 10.1038/s41467-021-23219-4 34016972 PMC8137702

[DEV200113C30] Hu, B. H., Zhang, C. and Frye, M. D. (2018). Immune cells and non-immune cells with immune function in mammalian cochleae. *Hear. Res.* 362, 14-24. 10.1016/j.heares.2017.12.009 29310977 PMC5911222

[DEV200113C31] Janesick, A. S. and Heller, S. (2019). Stem cells and the bird cochlea—where is everybody? *Cold Spring Harb. Perspect. Med.* 9, a033183. 10.1101/cshperspect.a033183 30249599 PMC6444699

[DEV200113C32] Janesick, A., Scheibinger, M., Benkafadar, N., Kirti, S., Ellwanger, D. C. and Heller, S. (2021). Cell-type identity of the avian cochlea. *Cell Rep.* 34, 108900. 10.1016/j.celrep.2021.108900 33761346

[DEV200113C33] Janesick, A., Scheibinger, M. and Heller, S. (2022). Molecular tools to study regeneration of the avian cochlea and utricle. In *Developmental, Physiological, and Functional Neurobiology of the Inner Ear* (ed. A. K. Groves), pp. 77-97. New York, NY: Springer US.

[DEV200113C34] Jassal, B., Matthews, L., Viteri, G., Gong, C., Lorente, P., Fabregat, A., Sidiropoulos, K., Cook, J., Gillespie, M., Haw, R. et al. (2019). The reactome pathway knowledgebase. *Nucleic Acids Res.* 48, D498-D503. 10.1093/nar/gkz1031 PMC7145712 31691815

[DEV200113C35] Jiang, H., Patel, P. H., Kohlmaier, A., Grenley, M. O., McEwen, D. G. and Edgar, B. A. (2009). Cytokine/Jak/Stat signaling mediates regeneration and homeostasis in the Drosophila midgut. *Cell* 137, 1343-1355. 10.1016/j.cell.2009.05.014 19563763 PMC2753793

[DEV200113C36] Julier, Z., Park, A. J., Briquez, P. S. and Martino, M. M. (2017). Promoting tissue regeneration by modulating the immune system. *Acta Biomater.* 53, 13-28. 10.1016/j.actbio.2017.01.056 28119112

[DEV200113C37] Kaur, T., Zamani, D., Tong, L., Rubel, E. W., Ohlemiller, K. K., Hirose, K. and Warchol, M. E. (2015). Fractalkine signaling regulates macrophage recruitment into the cochlea and promotes the survival of spiral ganglion neurons after selective hair cell lesion. *J. Neurosci.* 35, 15050-15061. 10.1523/JNEUROSCI.2325-15.2015 26558776 PMC4642237

[DEV200113C38] Ladrech, S., Wang, J., Simonneau, L., Puel, J.-L. and Lenoir, M. (2007). Macrophage contribution to the response of the rat organ of Corti to amikacin. *J. Neurosci. Res.* 85, 1970-1979. 10.1002/jnr.21335 17497672

[DEV200113C39] Li, X.-J. and Doetzlhofer, A. (2020). LIN28B/let-7 control the ability of neonatal murine auditory supporting cells to generate hair cells through mTOR signaling. *Proc. Natl. Acad. Sci. USA* 117, 22225-22236. 10.1073/pnas.2000417117 32826333 PMC7486708

[DEV200113C40] Liang, J., Wang, D., Renaud, G., Wolfsberg, T. G., Wilson, A. F. and Burgess, S. M. (2012). The stat3/socs3a pathway is a key regulator of hair cell regeneration in Zebrafish stat3/socs3a pathway: regulator of hair cell regeneration. *J. Neurosci.* 32, 10662-10673. 10.1523/JNEUROSCI.5785-10.2012 22855815 PMC3427933

[DEV200113C41] Lin, X., Zhou, Q., Zhao, C., Lin, G., Xu, J. and Wen, Z. (2019). An ectoderm-derived myeloid-like cell population functions as antigen transporters for langerhans cells in Zebrafish epidermis. *Dev. Cell* 49, 605-617.e5. 10.1016/j.devcel.2019.03.028 31006648

[DEV200113C42] Livak, K. J. and Schmittgen, T. D. (2001). Analysis of relative gene expression data using real-time quantitative PCR and the 2−ΔΔCT method. *Methods* 25, 402-408. 10.1006/meth.2001.1262 11846609

[DEV200113C43] Longfellow, H. W. (1882). *Elegiac Verse*.

[DEV200113C44] Malakhova, O. A., Kim, K. I. I., Luo, J.-K., Zou, W., Kumar, K. G. S., Fuchs, S. Y., Shuai, K. and Zhang, D.-E. (2006). UBP43 is a novel regulator of interferon signaling independent of its ISG15 isopeptidase activity. *EMBO J.* 25, 2358-2367. 10.1038/sj.emboj.7601149 16710296 PMC1478183

[DEV200113C45] Martín-Vicente, M., Medrano, L. M., Resino, S., García-Sastre, A. and Martínez, I. (2017). TRIM25 in the regulation of the antiviral innate immunity. *Front. Immunol.* 8, 1187. 10.3389/fimmu.2017.01187 29018447 PMC5614919

[DEV200113C46] Matsunaga, M., Kita, T., Yamamoto, R., Yamamoto, N., Okano, T., Omori, K., Sakamoto, S. and Nakagawa, T. (2020). Initiation of supporting cell activation for hair cell regeneration in the avian auditory epithelium: an explant culture model. *Front. Cell. Neurosci.* 14, 583994. 10.3389/fncel.2020.583994 33281558 PMC7688741

[DEV200113C87] McCarthy, D. J., Campbell, K. R., Lun, A. T. and Wills, Q. F. (2017). Scater: pre-processing, quality control, normalization and visualization of single-cell RNA-seq data in R. *Bioinformatics* 33, 1179-1186. 10.1093/bioinformatics/btw777 28088763 PMC5408845

[DEV200113C47] McElrath, C., Espinosa, V., Lin, J.-D., Peng, J., Sridhar, R., Dutta, O., Tseng, H.-C., Smirnov, S. V., Risman, H., Sandoval, M. J. et al. (2021). Critical role of interferons in gastrointestinal injury repair. *Nat. Commun.* 12, 2624. 10.1038/s41467-021-22928-0 33976143 PMC8113246

[DEV200113C49] Monzack, E. L. and Cunningham, L. L. (2013). Lead roles for supporting actors: Critical functions of inner ear supporting cells. *Hear. Res.* 303, 20-29. 10.1016/j.heares.2013.01.008 23347917 PMC3648608

[DEV200113C88] Nagai, H., Sezaki, M., Bertocchini, F., Fukuda, K. and Sheng, G. (2014). HINTW, a W-chromosome HINT gene in chick, is expressed ubiquitously and is a robust female cell marker applicable in intraspecific chimera studies. *Genesis* 52, 424-430. 10.1002/dvg.22769 24599776

[DEV200113C50] Nagao, M., Ogata, T., Sawada, Y. and Gotoh, Y. (2016). Zbtb20 promotes astrocytogenesis during neocortical development. *Nat. Commun.* 7, 11102. 10.1038/ncomms11102 27000654 PMC4804180

[DEV200113C51] Neerukonda, S. N. and Katneni, U. (2020). Avian pattern recognition receptor sensing and signaling. *Vet. Sci.* 7, 14. 10.3390/vetsci7010014 32012730 PMC7157566

[DEV200113C52] Nguyen, A. V. and Soulika, A. M. (2019). The dynamics of the skin's immune system. *Int. J. Mol. Sci.* 20, 1811. 10.3390/ijms20081811 31013709 PMC6515324

[DEV200113C53] O'Halloran, E. K. and Oesterle, E. C. (2004). Characterization of leukocyte subtypes in chicken inner ear sensory epithelia. *J. Comp. Neurol.* 475, 340-360. 10.1002/cne.20162 15221950

[DEV200113C54] Orvis, J., Gottfried, B., Kancherla, J., Adkins, R. S., Song, Y., Dror, A. A., Olley, D., Rose, K., Chrysostomou, E., Kelly, M. C. et al. (2021). gEAR: Gene expression analysis resource portal for community-driven, multi-omic data exploration. *Nat. Methods* 18, 843-844. 10.1038/s41592-021-01200-9 34172972 PMC8996439

[DEV200113C55] Picelli, S., Faridani, O. R., Björklund, Å. K., Winberg, G., Sagasser, S. and Sandberg, R. (2014). Full-length RNA-seq from single cells using Smart-seq2. *Nat. Protoc.* 9, 171-181. 10.1038/nprot.2014.006 24385147

[DEV200113C57] Quintás-Cardama, A., Vaddi, K., Liu, P., Manshouri, T., Li, J., Scherle, P. A., Caulder, E., Wen, X., Li, Y., Waeltz, P. et al. (2010). Preclinical characterization of the selective JAK1/2 inhibitor INCB018424: therapeutic implications for the treatment of myeloproliferative neoplasms. *Blood* 115, 3109-3117. 10.1182/blood-2009-04-214957 20130243 PMC3953826

[DEV200113C58] Rai, V., Wood, M. B., Feng, H., Schabla, N. M., Tu, S. and Zuo, J. (2020). The immune response after noise damage in the cochlea is characterized by a heterogeneous mix of adaptive and innate immune cells. *Sci. Rep.* 10, 15167. 10.1038/s41598-020-72181-6 32938973 PMC7495466

[DEV200113C59] Raphael, Y., Kim, Y.-H., Osumi, Y. and Izumikawa, M. (2007). Non-sensory cells in the deafened organ of Corti: approaches for repair. *Int. J. Dev. Biol.* 51, 649-654. 10.1387/ijdb.072370yr 17891723

[DEV200113C60] Rau, A., Gallopin, M., Celeux, G. and Jaffrézic, F. (2013). Data-based filtering for replicated high-throughput transcriptome sequencing experiments. *Bioinformatics* 29, 2146-2152. 10.1093/bioinformatics/btt350 23821648 PMC3740625

[DEV200113C61] Robinson, M. D. and Oshlack, A. (2010). A scaling normalization method for differential expression analysis of RNA-seq data. *Genome Biol.* 11, R25. 10.1186/gb-2010-11-3-r25 20196867 PMC2864565

[DEV200113C62] Robinson, M. D., McCarthy, D. J. and Smyth, G. K. (2010). edgeR: a Bioconductor package for differential expression analysis of digital gene expression data. *Bioinformatics* 26, 139-140. 10.1093/bioinformatics/btp616 19910308 PMC2796818

[DEV200113C63] Ryals, B. M., Dent, M. L. and Dooling, R. J. (2013). Return of function after hair cell regeneration. *Hear. Res.* 297, 113-120. 10.1016/j.heares.2012.11.019 23202051 PMC3593961

[DEV200113C64] Santhakumar, D., Rubbenstroth, D., Martinez-Sobrido, L. and Munir, M. (2017). Avian interferons and their antiviral effectors. *Front. Immunol.* 8, 49. 10.3389/fimmu.2017.00049 28197148 PMC5281639

[DEV200113C65] Saunders, J. C. and Salvi, R. J. (2008). Recovery of Function in the Avian Auditory System After Ototrauma. In *Hair Cell Regeneration, Repair, and Protection* (ed. R. J. Salvi, A. N. Popper and R. R. Fay), pp. 77-116. New York, NY: Springer New York.

[DEV200113C66] Scheibinger, M., Ellwanger, D. C., Corrales, C. E., Stone, J. S. and Heller, S. (2018). Aminoglycoside damage and hair cell regeneration in the chicken utricle. *J. Assoc. Res. Otolaryngol.* 19, 17-29. 10.1007/s10162-017-0646-4 29134476 PMC5783928

[DEV200113C67] Scheibinger, M., Janesick, A., Benkafadar, N., Ellwanger, D. C., Jan, T. A. and Heller, S. (2021). Cell-type identity of the avian utricle. *SSRN Electron. J*. 10.2139/ssrn.3831459 PMC9588199 36170825

[DEV200113C68] Scheibinger, M., Janesick, A., Diaz, G. H. and Heller, S. (2022). Immunohistochemistry and In Situ mRNA detection using inner ear vibratome sections. In *Developmental, Physiological, and Functional Neurobiology of the Inner Ear* (ed. A. K. Groves), pp. 41-58. New York, NY: Springer US.

[DEV200113C69] Schwaller, B. (2009). The continuing disappearance of “pure” Ca2+ buffers. *Cell. Mol. Life Sci.* 66, 275-300. 10.1007/s00018-008-8564-6 19099190 PMC11131537

[DEV200113C70] Schwaller, B. and Herrmann, B. (1997). Regulated redistribution of calretinins in WiDr cells. *Cell Death Differ.* 4, 325-333. 10.1038/sj.cdd.4400240 16465248

[DEV200113C71] Shrestha, B. R., Chia, C., Wu, L., Kujawa, S. G., Liberman, M. C. and Goodrich, L. V. (2018). Sensory neuron diversity in the inner ear is shaped by activity. *Cell* 174, 1229-1246.e17. 10.1016/j.cell.2018.07.007 30078709 PMC6150604

[DEV200113C72] Stone, J. S. and Rubel, E. W. (1999). Delta1 expression during avian hair cell regeneration. *Development* 126, 961-973. 10.1242/dev.126.5.961 9927597

[DEV200113C73] Su, S., Law, C. W., Ah-Cann, C., Asselin-Labat, M.-L., Blewitt, M. E. and Ritchie, M. E. (2017). Glimma: interactive graphics for gene expression analysis. *Bioinformatics* 33, 2050-2052. 10.1093/bioinformatics/btx094 28203714 PMC5870845

[DEV200113C74] Sun, L., Miyoshi, H., Origanti, S., Nice, T. J., Barger, A. C., Manieri, N. A., Fogel, L. A., French, A. R., Piwnica-Worms, D., Piwnica-Worms, H. et al. (2015). Type I interferons link viral infection to enhanced epithelial turnover and repair. *Cell Host Microbe* 17, 85-97. 10.1016/j.chom.2014.11.004 25482432 PMC4297260

[DEV200113C75] Sun, S., Babola, T., Pregernig, G., So, K. S., Nguyen, M., Su, S.-S. M., Palermo, A. T., Bergles, D. E., Burns, J. C. and Müller, U. (2018). Hair cell mechanotransduction regulates spontaneous activity and spiral ganglion subtype specification in the auditory system. *Cell* 174, 1247-1263.e15. 10.1016/j.cell.2018.07.008 30078710 PMC6429032

[DEV200113C76] Tahara, E., Tahara, H., Kanno, M., Naka, K., Takeda, Y., Matsuzaki, T., Yamazaki, R., Ishihara, H., Yasui, W., Barrett, J. C. et al. (2005). G1P3, an interferon inducible gene 6-16, is expressed in gastric cancers and inhibits mitochondrial-mediated apoptosis in gastric cancer cell line TMK-1 cell. *Cancer Immunol. Immunother.* 54, 729-740. 10.1007/s00262-004-0645-2 15685448 PMC11034321

[DEV200113C77] Tornabene, S. V., Sato, K., Pham, L., Billings, P. and Keithley, E. M. (2006). Immune cell recruitment following acoustic trauma. *Hear. Res.* 222, 115-124. 10.1016/j.heares.2006.09.004 17081714

[DEV200113C78] Tucci, D. L. and Rubel, E. W. (1990). Physiologic status of regenerated hair cells in the avian inner ear following aminoglycoside ototoxicity. *Otolaryngol. Neck Surg.* 103, 443-450. 10.1177/019459989010300317 2122376

[DEV200113C79] Ulgen, E., Ozisik, O. and Sezerman, O. U. (2019). pathfindR: an R Package for comprehensive identification of enriched pathways in omics data through active subnetworks. *Front. Genet.* 10, 858. 10.3389/fgene.2019.00858 31608109 PMC6773876

[DEV200113C80] Wan, G., Corfas, G. and Stone, J. S. (2013). Inner ear supporting cells: Rethinking the silent majority. *Semin. Cell Dev. Biol.* 24, 448-459. 10.1016/j.semcdb.2013.03.009 23545368 PMC4005836

[DEV200113C81] Warchol, M. (1997). Macrophage activity in organ cultures of the avian cochlea: demonstration of a resident population and recruitment to sites of hair cell lesions. *J. Neurobiol.* 33, 724-734. 10.1002/(SICI)1097-4695(19971120)33:6<724::AID-NEU2>3.0.CO;2-B 9369147

[DEV200113C82] Warchol, M. E., Schwendener, R. A. and Hirose, K. (2012). Depletion of resident macrophages does not alter sensory regeneration in the avian cochlea. *PLoS ONE* 7, e51574. 10.1371/journal.pone.0051574 23240046 PMC3519890

[DEV200113C83] Warchol, M. E., Schrader, A. and Sheets, L. (2021). Macrophages respond rapidly to ototoxic injury of lateral line hair cells but are not required for hair cell regeneration. *Front. Cell. Neurosci.* 14, 613246. 10.3389/fncel.2020.613246 33488362 PMC7820375

[DEV200113C84] Wilmes, S., Beutel, O., Li, Z., Francois-Newton, V., Richter, C. P., Janning, D., Kroll, C., Hanhart, P., Hötte, K., You, C. et al. (2015). Receptor dimerization dynamics as a regulatory valve for plasticity of type I interferon signaling. *J. Cell Biol.* 209, 579-593. 10.1083/jcb.201412049 26008745 PMC4442803

[DEV200113C85] Wood, M. B. and Zuo, J. (2017). The contribution of immune infiltrates to ototoxicity and cochlear hair cell loss. *Front. Cell. Neurosci.* 11, 106. 10.3389/fncel.2017.00106 28446866 PMC5388681

[DEV200113C86] Zheng, J. L. and Gao, W.-Q. (1997). Analysis of rat vestibular hair cell development and regeneration using calretinin as an early marker. *J. Neurosci.* 17, 8270-8282. 10.1523/JNEUROSCI.17-21-08270.1997 9334402 PMC6573764

