# Supplementary material for: Avian auditory hair cell regeneration is accompanied by JAK/STAT-dependent expression of immune-related genes in supporting cells
Source: Development. 2022 May 3;149(8):dev200113. doi: 10.1242/dev.200113 (PMC10656459; doi:10.1242/dev.200113)
Supplement: Supplementary information [file develop-149-200113-s1.pdf]

Supplemental Figure S1

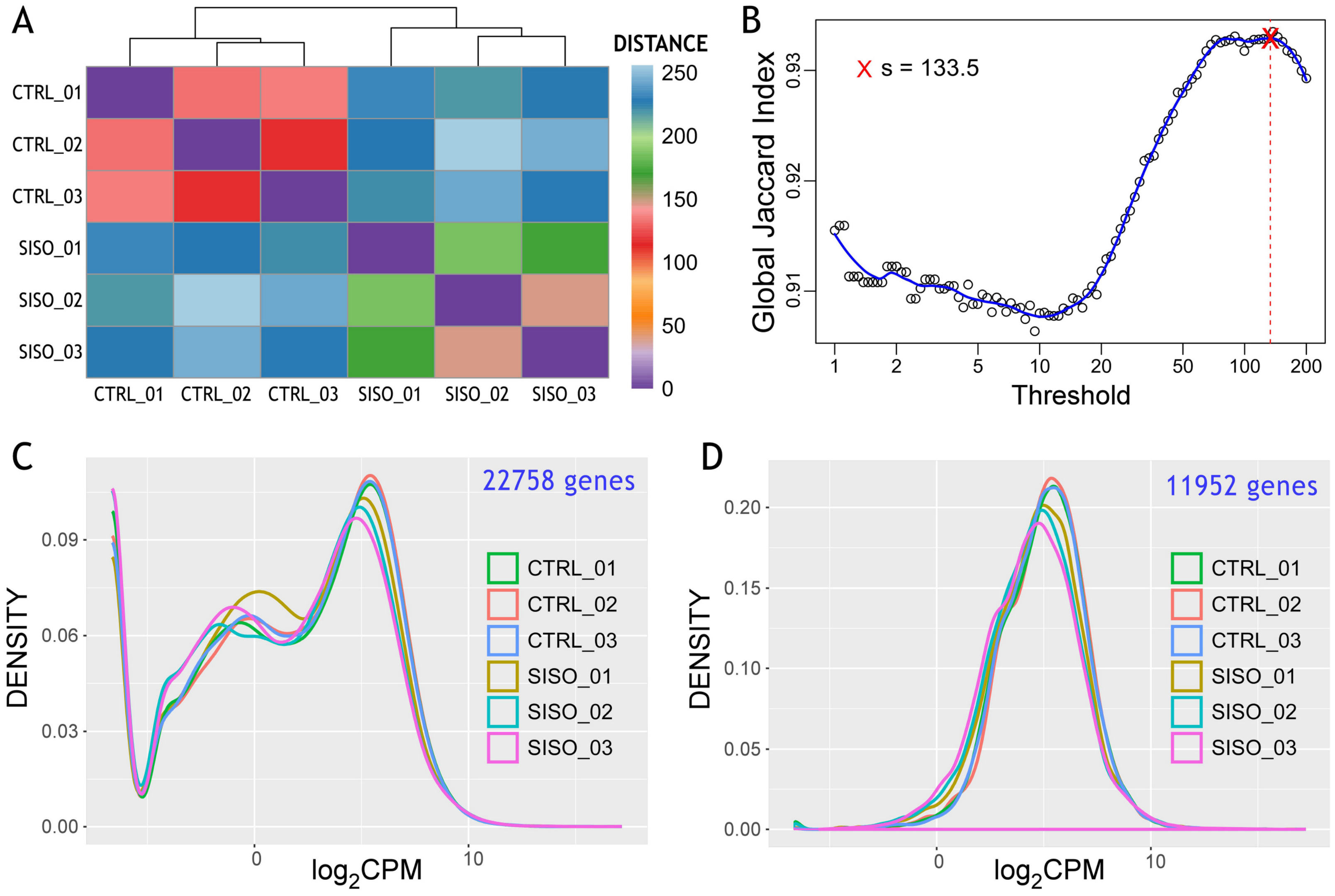

Fig. S1. Quality control, filtering and normalization of bulk RNA-seq data. (A) Sample-to-sample correlation/ distance matrix (units = log_2(counts+l)) demonstrate that sisomicin and contralateral controls cluster in separate groups. **(B)** Calculation of the Jaccard Index using the HTSFilter package. Genes with TMM normalized counts less than or equal to 133.5 are removed. **(C, D)** Geometric density plots visualize the distribution of log_2(Counts-Per-Million)values before **(C)** and after **(D)** gene filtering (by HTSFilter) and normalization (TMM = trimmed-mean-of-M-values).

Development • Supplementary information

Supplemental Figure S2

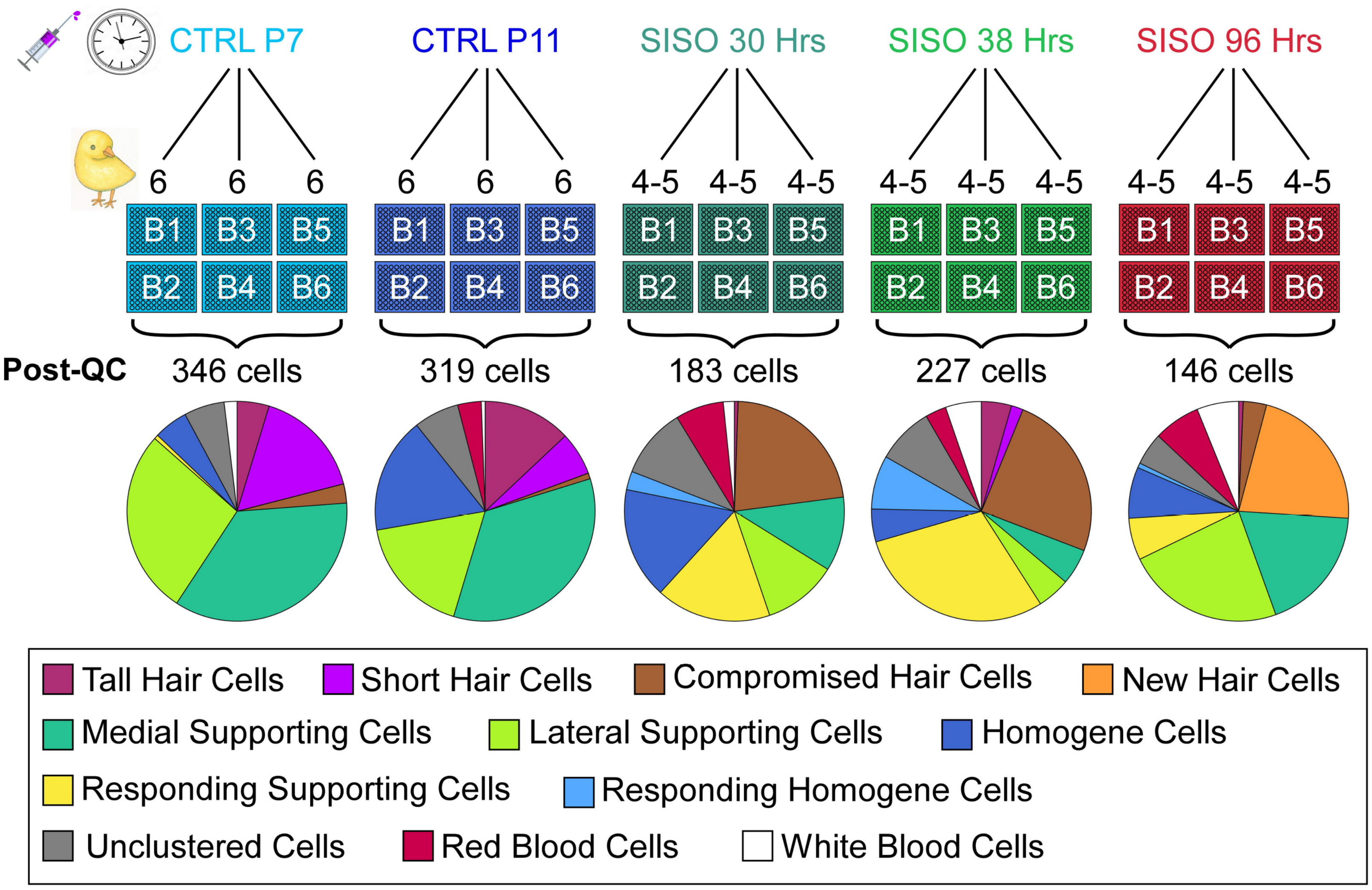

Fig. S2. Experimental design for single cell RNA-seq. Timepoints (either control or surgery) are listed at the top. For timepoints requiring surgery (denoted as SISO = sisomicin infusion), we would begin with 6-7 animals. Because we can grossly detect whether a peeled sensory epithelium is completely damaged, we would select the best (most damaged and intact) 4-5 out of the 6-7 animals to be pooled for further processing (dissociation, FACS, and sequencing). For control animals (CTRL), all epithelium collected were usable (N = 6, pooled). Two 96-well plates were loaded using FACS from the pooled epithelia (e.g., "B1 and B2" = batch 1 and batch 2). In order to maintain biological variability, we collected pooled epithelia on three separate weeks (three different clutches of chickens). Therefore, N = 3 (B1/2, B3/4, and B5/6) yields six 96-well plates for each timepoint. After quality control (cDNA bioanalyzing and bioinformatic filtering), we were left with the number of cells reported (post-QC #). After dimension reduction, clustering, and marker gene assignment, we can designate an ID to each cell. The proportions of each cell ID are displayed as pie charts. Note that "unclustered" cells (which we could not assign to a specific cell type) are not displayed in the tsne plots of all the main figures.

Development • Supplementary information

Supplemental Figure S3

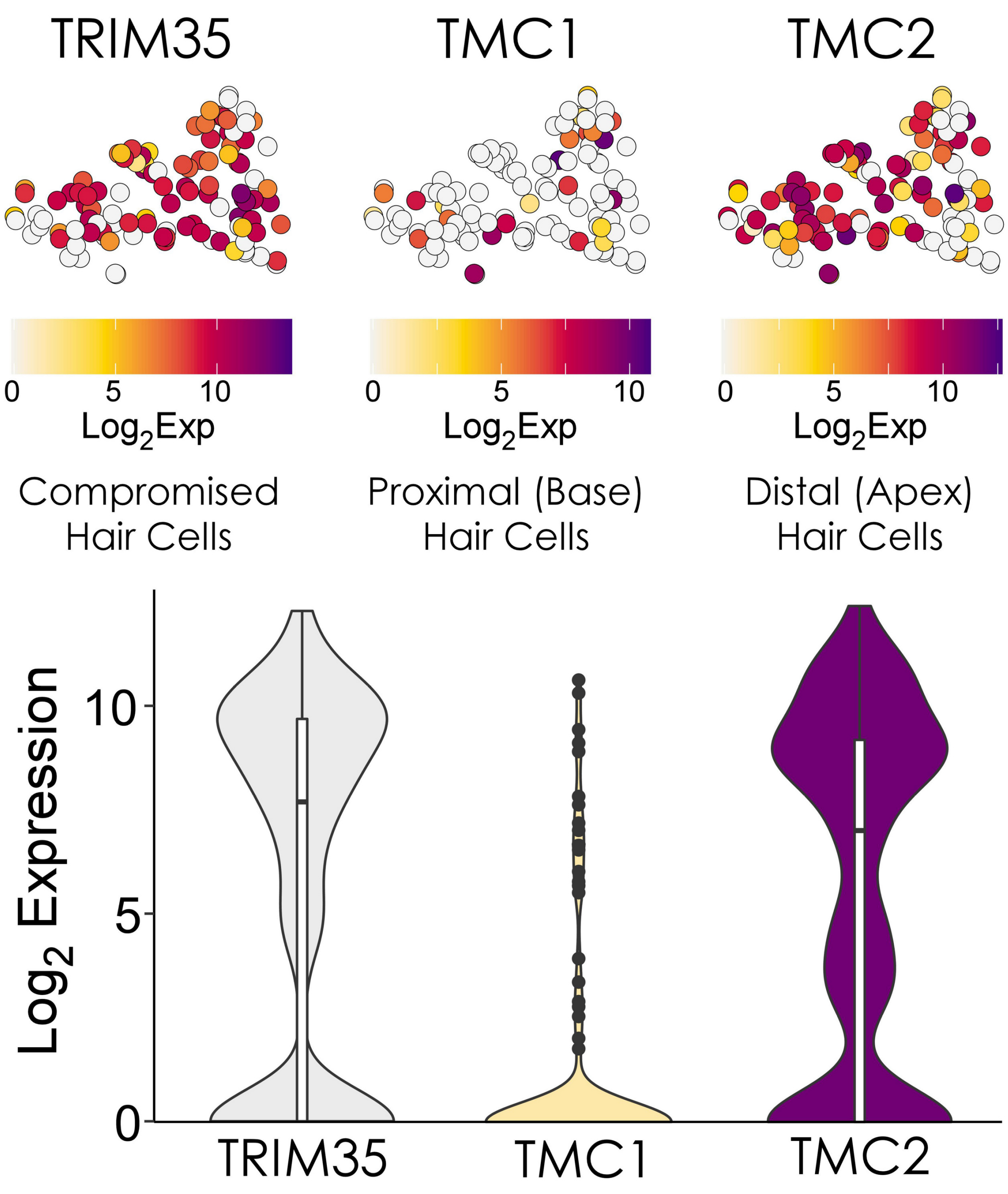

Fig. S3. Compromised hair cells are proximal cells. tSNE and violin plots displaying $\log_2$ transformed, normalized expression counts for *TRIM35*, *TMC1*, and *TMC2*, showing only compromised hair cells. Proximal hair cells are mostly absent (damaged and ejected) as indicated by lower expression of *TMC1*. Distal hair cells, marked by *TMC2*, are more resilient and survive.

Supplemental Figure 4

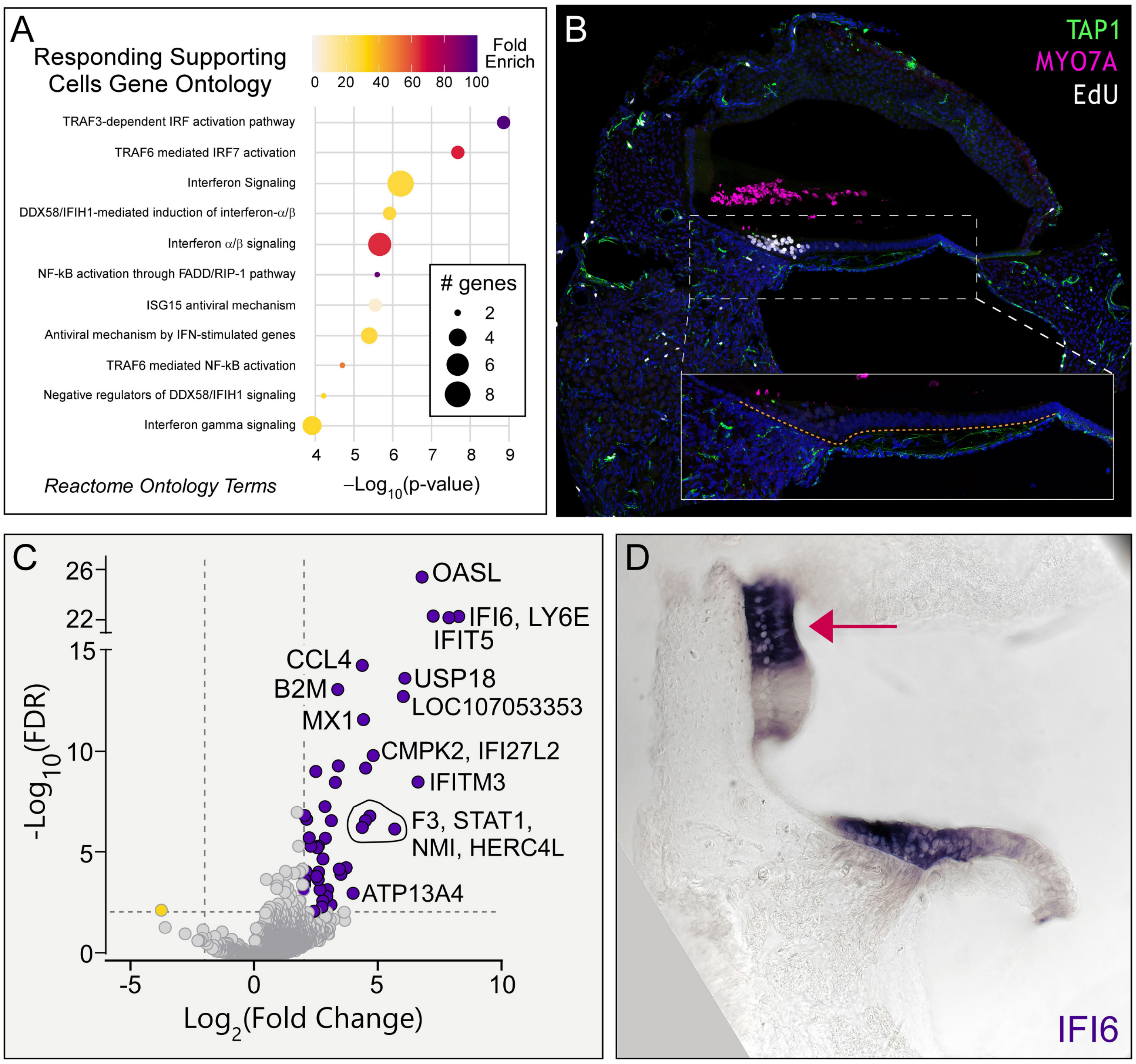

Fig. S4. Additional characterization of responding supporting cells and homogene cells. (A) Gene ontology analysis of genes up-regulated in the responding supporting cell group using pathfindR analysis tool and Reactome database. **(B)** Immunohistochemistry for TAP1 in green (macrophages), MY07A in magenta (hair cell corpses), EdU in white (proliferating supporting cells), and Dapi in blue (cell nuclei). Inset shows the same specimen with the EdU channel to demonstrate that TAPI expression is absent from supporting cells. Dotted orange line marks the basement membrane. **(C)** Volcano plot illustrating genes expressed in the responding homogene cell group (purple dots) at least 4-fold higher and significantly different (FDR < 0.01) compared with genes expressed in non-responding homogene cells (yellow dots). **(D)** In situ hybridization validates *IF16* mRNA expression in a subgroup of homogene cells (red arrow; known as "top homogene" in Janesick et al., 2021b) in P9 transverse sections, 48 hours post-sisomicin damage.

Supplemental Figure S5

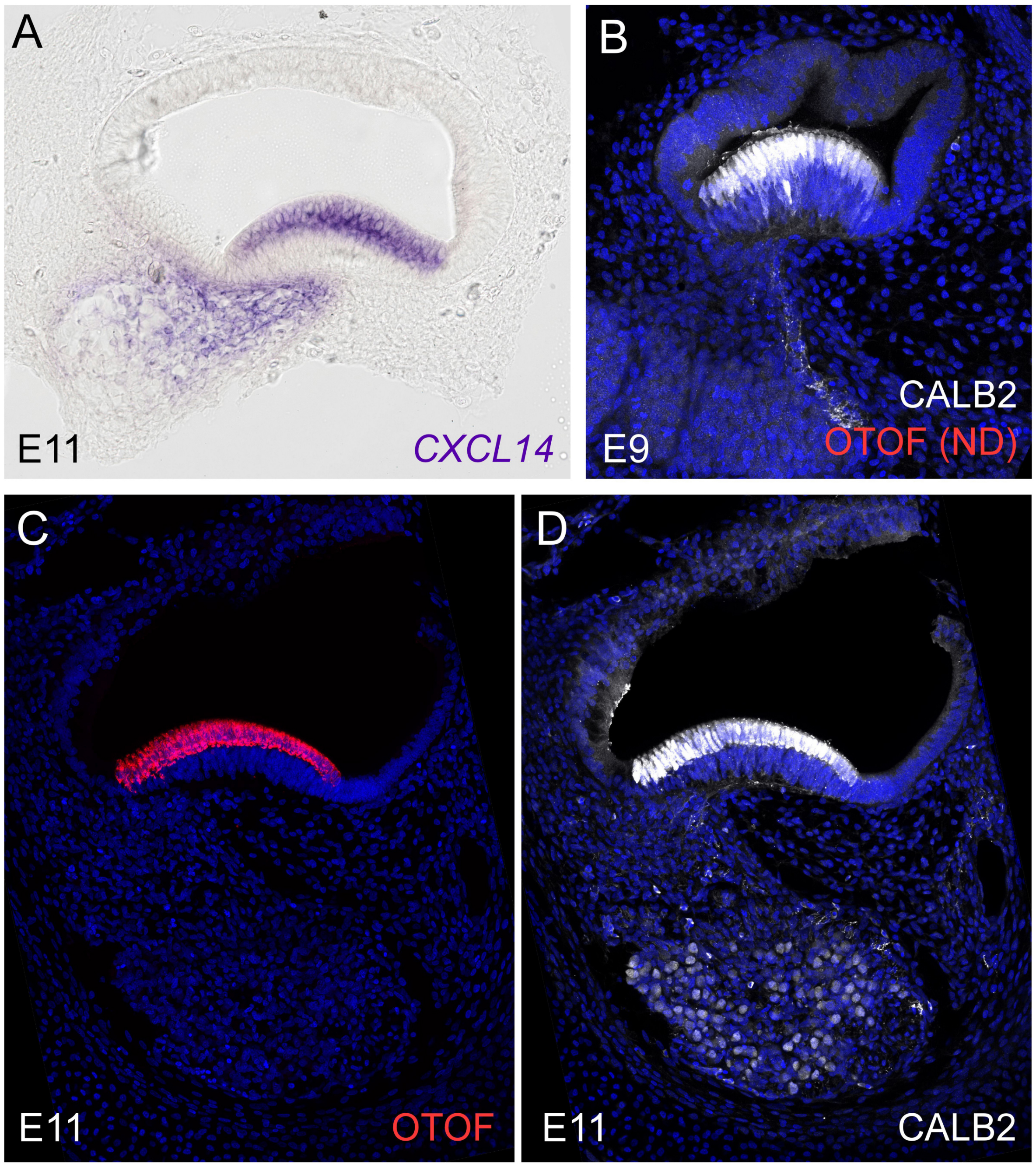

Fig. S5. Developmental expression of *CXCL14*, CALB2, and OTOF. (A) In situ hybridization in vibratome sections through the E11 cochlea duct, staining for *CXCL14* mRNA. (B) Immunohistochemistry for CALB2 (a.k.a. Calretinin) at E9. OTOF is not detected (ND) at this stage. **(C-D**) Immunohistochemistry detects OTOF **(C)** and CALB2 **(D)** at E11.

Table S1. Literature references of past transcriptomics studies of regenerating avian BP and utricle.

Click here to download Table S1

Table S2. Bulk RNA-Sequencing dataset (2 days post-sisomicin): Differential expression analysis with EdgeR.

Click here to download Table S2

Table S3. Single Cell RNA-Seq: Cell identity counts for pie charts shown in Supplemental Figure S2.

Click here to download Table S3

Table S4. Distinguishing markers of responding supporting cells, corresponds to Figure 3A.

Click here to download Table S4

Table S5. Distinguishing markers of responding homogene cells, corresponds to Figure S4C.

Click here to download Table S5

Table S6. Distinguishing markers of new versus mature hair cells.

Click here to download Table S6

Table S7. Bulk RNA-Sequencing dataset (2 days post-sisomicin): Raw count matrix (pre-normalization, pre-filtering).

Click here to download Table S7

Table S8. In situ hybridization primer sets used to synthesize probes in Figures 3D-F, 6B', 6D', and Figures S4D, S5A.

Click here to download Table S8

Table S9. Antibodies used for immunofluorescence images in Figures 4, 6E-G', S4B, S5B-D

Click here to download Table S9

Table S10. QPCR primer sets used in Figure 5.

Click here to download Table S10
