# Supplementary material for: Avian auditory hair cell regeneration is accompanied by JAK/STAT-dependent expression of immune-related genes in supporting cells
Source: Development. 2022 May 3;149(8):dev200113. doi: 10.1242/dev.200113 (PMC10656459; doi:10.1242/dev.200113)
Supplement: Supplementary file 1 [file develop-149-200113_review_history.pdf]

Avian auditory hair cell regeneration is accompanied by JAK/STAT-dependent expression of immune-related genes in supporting cells

Amanda S. Janesick, Mirko Scheibinger, Nesrine Benkafadar, Sakin Kirti and Stefan Heller
DOI: 10.1242/dev.200113

Editor: Paul Martin

Review timeline

Original submission:	23 September 2021
Editorial decision:	3 November 2021
First revision received:	23 December 2021
Editorial decision:	2 February 2022
Second revision received:	4 March 2022
Accepted:	31 March 2022

Original submission

First decision letter

MS ID#: DEVELOP/2021/200113

MS TITLE: Chicken Auditory Supporting Cells Express Interferon Response Genes during Regeneration towards Nascent Sensory Hair Cells In Vivo

AUTHORS: Amanda S Janesick, Mirko Scheibinger, Nesrine Benkafadar, Sakin Kirti, and Stefan Heller

I have now received all the referees' reports on the above manuscript, and have reached a decision. The referees' comments are appended below, or you can access them online: please go to BenchPress and click on the 'Manuscripts with Decisions' queue in the Author Area.

As you will see, the two expert referees express considerable interest in your work, but have some significant criticisms and recommend a substantial revision of your manuscript before we can consider publication. If you are able to revise the manuscript along the lines suggested, which I think will necessitate further experiments, I will be happy receive a revised version of the manuscript. Your revised paper will be re-reviewed by one or more of the original referees, and acceptance of your manuscript will depend on your addressing satisfactorily the reviewers' major concerns. Please also note that Development will normally permit only one round of major revision.

We are aware that you may be experiencing disruption to the normal running of your lab that make experimental revisions challenging. If it would be helpful, we encourage you to contact us to discuss your revision in greater detail. Please send us a point-by-point response indicating where you are able to address concerns raised (either experimentally or by changes to the text) and where you will not be able to do so within the normal timeframe of a revision. We will then provide further guidance. Please also note that we are happy to extend revision timeframes as necessary.

Please attend to all of the reviewers' comments and ensure that you clearly highlight all changes made in the revised manuscript. Please avoid using 'Tracked changes' in Word files as these are lost in PDF conversion. I should be grateful if you would also provide a point-by-point response detailing how you have dealt with the points raised by the reviewers in the 'Response to Reviewers' box. If you do not agree with any of their criticisms or suggestions please explain clearly why this is so.

© 2022. Published by The Company of Biologists under the terms of the Creative Commons Attribution License (https://creativecommons.org/licenses/by/4.0/).

Reviewer 1

Advance summary and potential significance to field

The manuscript of Janesick et al. concerns the mechanisms that regulate the regeneration of sensory receptors in the avian inner ear. The manuscript has three primary foci, which are not entirely interrelated. First, the authors describe scRNA-Seq data of exceptionally high quality, demonstrating that numerous interferon signaling genes are expressed in response to hair cell injury. The second (somewhat related) focus is on the role of JAK/STAT signaling in regeneration. Using an epithelial culture system, it is demonstrated that inhibition of JAK leads to a reduction in regenerative proliferation, as well as decreased expression of interferon (INF) signaling genes. This part of the manuscript raises several questions that are not addressed. The third focus of this paper concerns the expression of calretinin (CALB2) is the regenerating basilar papilla. This issue is not explored in any detail and is not directly related to the other data reported in this manuscript.

Comments for the author

The manuscript of Janesick et al. has three primary foci, which are not entirely interrelated. First, the authors describe scRNA-Seq data of exceptionally high quality, demonstrating that numerous interferon signaling genes are expressed in response to hair cell injury. Those results are largely novel. Although (as acknowledged) some similar results have been reported by Matsunaga et al. (Front Neurosci, 2020), the present data are much more complete and thoroughly verified. As such, these data are a highly important contribution to the study of otic regeneration and implicate a role for inflammation (and inflammatory genes) in that process.
The second (somewhat related) focus is on the role of JAK/STAT signaling in regeneration. Using an epithelial culture system, it is demonstrated that inhibition of JAK leads to a reduction in regenerative proliferation, as well as decreased expression of interferon (INF) signaling genes. This part of the manuscript raises several questions that are not addressed. First, what is the precise relationship between JAK activation and INF signaling? It is commonly thought that JAK activation occurs downstream of INF activation (e.g., Mazewski et al., Front Immunol 2020). Are the authors suggesting that IFN triggers JAK/STAT, which then leads to expression of IFN signaling genes? The authors need to clarify this point. Also (as acknowledged in the text), JAK can be activated by a number of upstream signals. I think that identifying those signal/signals in the damaged cochlea. would be of critical importance. It is also possible that initial hair cell damage causes activation of IFN, which then causes activation of JAK/STAT, which then promotes expression of IFN target genes, thereby initiating a positive feedback loop that leads to further JAK activation, IFN gene expression, etc. It is notable that such a process would be blocked by RUX treatment that occurs shortly after injury. To summarize: the demonstration that IFN signaling is activated after injury is a very interesting and important observation. More attention should be given to identifying the steps in that pathway that induced and regulate this activation. Finally, what is the role of IFN gene expression in the process of regeneration? This presented data do not demonstrate that the downstream IFN genes are essential.
It is conceivable that IFN signaling is activated by tissue damage, but that it plays NO role in triggering regeneration. If that were the case, the gene expression results described above would be of lesser interest. The presented data are very strong, but the authors need to clarify the causal link between IFN, JAK/STAT and hair cell regeneration.
The authors should also provide some rationale for use of the epithelial culture system. It could be argued that isolated epithelia, adhering to a glass substrate via Cell Tak, are not an optimal model for these experiments. The native environment (e.g., ECM proteins, etc.) provided by organotypic cultures may be more realistic. I am not requesting that the authors repeat these studies, but the pro's and con's of the epithelial culture system should be discussed.
The third focus of this paper concerns the expression of calretinin (CALB2) is the regenerating basilar papilla. The authors show expression of CALB2 in both regenerative supporting cells as well as in replacement hair cells. The supporting cell expression is novel and likely to be interesting. However, this issue is not explored in any detail and is not directly related to the other data reported in this manuscript. I recommend omitting those data from the present paper and publishing them once a more complete story has been obtained.
The demonstration of inflammatory signaling is of great interest and should really be the focus of this paper.

© 2022. Published by The Company of Biologists under the terms of the Creative Commons Attribution License (https://creativecommons.org/licenses/by/4.0/).

In summary, the authors have performed an outstanding genomic characterization of the regenerating chick cochlea, which resulted in the very intriguing suggestion that IFN signaling may be involved in the regenerative process. The study requires a more mechanistic picture of the role of IFN signaling in the regenerative process.

Minor issues:
(1) The authors appear confident about the purity of their cell isolation system, i.e., that it yields only cells from the sensory epithelium of the basilar papilla. If this is true, then we are there small clusters of red and white blood cells in the tSNE plots shown in Fig. 2?
(2) Does hair cell production in the superior region of the papilla occur via symmetric or asymmetric division (or both)? If it occurred vis asymmetric proliferation, then we would expect to see approximately equal numbers of labeled hair cells and supporting cells. In fact, they differ (87% vs. 59%).
(3) The discussion of phalangeal scaring in the cochlea is somewhat misleading.
The data in the cited paper (Lee et al., Neurosci 2021) do not show clear evidence for MET in the damaged cochlea. Instead, that study characterized the process of epithelial repair (via rearrangement at the lumen), and also that Deiters cells could expand and engulf prestin-labeled debris of dead OHC's. The results of Hu and Corwin (2007) are derived from cultures of isolated supporting cells and their relevance to repair in vivo are not clear. In any case, the use of the term 'scar' to describe this process is unfortunate, because it implies certain similarities between epithelial repair in the inner ear and epidermal wound healing. Epithelial repair in the ear is (usually) quite simple, i.e., adjoining supporting cells expand to reseal gaps in the epithelial barrier caused by the death of hair cells. Some supporting cells may also engulf cellular debris. However, except in extreme situations, repair to the cochlea does not involve EMT, MET or fibrosis. I acknowledge that the use of the term 'scar' to describe cochlear repair has been common for ~30 years and certainly did not originate with the present authors. But, because that term is applied to repair in the cochlea, it is not fair to assume that other features of tissue scarring in (for example) skin wounds also occur in the ear.
(4) In the Discussion, the author quote H.W. Longfellow, but no citation is given for this quotation. Such omission could be detrimental to Mr. Longfellow's h-index and diminish the significance of his literary career.

Reviewer 2

Advance summary and potential significance to field

This paper is the first to use single-cell RNA-Seq to analyse gene expression levels in the course of hair cell (HC) regeneration in the chicken basilar papilla (BP). This organ is an important model system for HC regeneration studies: following HC death, the supporting cells that surround them can give rise to new HC by direct transdifferentiation or by a mitotic route. The signals that initiate the regenerative response are unknown and transcriptome analyses such as those performed in this study could potentially shed new light on their nature.
Here, the authors use the intra inner ear injection of an aminoglysocide antibiotic to induce rapid, massive and synchronous HC death throughout the BP of young chicks. This protocol has been described by the authors in a recent study investigating the changes in gene expression during hair cell death in the BP. This study is the natural extension of this work, focusing on genes expressed within the regenerating epithelium in the days following hair cell death.
Their findings from bulk and single-cell RNA-seq datasets collected and analysed at different time points post-damage confirm recent work (Matsunaga et al. 2020) and show that i) the expression of several interferon response genes is upregulated in a subset of "responding" supporting cells (the progenitors for new hair cells) after hair cell damage and that ii) the JAK/STAT pathway is required for this response. The single-cell analyses reveal different clusters of supporting cells post-damage, which could represent cells engaging in different types of regenerative/proliferative responses; a new marker for regenerated hair cells, calretinin (CALB2), is also identified.
Finally, the paper describes the spatio-temporal pattern of hair cell regeneration and supporting cell proliferation in their ototoxicity model.

The main advance provided by this study is the single-cell RNA-Seq dataset itself, which represents a very valuable resource for the inner ear research community: the experiments are well designed

© 2022. Published by The Company of Biologists under the terms of the Creative Commons Attribution License (https://creativecommons.org/licenses/by/4.0/).

and executed, several time points post-damage are analysed, bulk versus single-cell RNA-Seq datasets are compared, and the bioinformatics analyses suggest high-quality, information-rich datasets.
On the other hand, the actual role of interferon signalling in the context of hair cell regeneration remains unclear, as is the potential role of the other genes singled-out by the authors in their bioinformatics analyses. The conceptual advances, as far as the molecular basis/triggers of hair cell regeneration is concerned, are therefore limited. Furthermore, the rationale and interpretation of some of the analyses trying to establish the “link” between responding supporting cells and the newly regenerated hair cells are unclear to me.

Comments for the author

Main comments:
- Role of JAK/STAT in HC regeneration. There is now strong evidence, based on the present study and the recent one by Matsunaga et al (2020) that interferon-response genes are upregulated in SC following HC death. The pharmacological inhibition of JAK/STAT activity with RUX in vitro (also previously performed by Matsunaga et al 2020) confirm the implication of this pathway in the upregulation of interferon-response genes in SC, but one wonders why the authors did not investigate the effects of RUX on hair cell regeneration itself. This would have allowed much stronger conclusions to be drawn from the study. I was also surprised to see in Fig. 5A that RUX induces a significant upregulation of the expression of HC-specific genes in control cultures; this result is not mentioned in the text of the legend/results, but it suggests to me that RUX could potentially impact on cell composition/transcription in the absence of damage. One can appreciate that a comprehensive study of the effects of RUX on HC regeneration may be beyond the scope of this study, but it seems that a simple immunostaining for HC/SC markers at the end of the RUX treatment in vitro could have helped to resolve some of these questions.
- Section “CALB2, USP18, and TRIM25 link responding supporting cells to new hair cells”. Early in this section, the authors write that: “Our next goal was to link the responding supporting cell population to new hair cells.”, which suggests a strong bias in their exploration of the data… the analyses themselves reinforce this sentiment and do not, in my view, support the conclusions of the authors:
“In lieu of a trajectory analysis, we compared the top-ranking genes of the new hair cell and the responding supporting cell group and found three genes present in both: CALB2, USP18, and TRIM25 (Fig. 6A-D).” What exactly are these comparisons? Are the top-ranking genes in these two datasets selected according to log2FC or FDR values? How many top-ranking genes from each dataset are compared? From a rapid survey of the supplementary files for the DEG in “responding SC” and “new HC”, I found that about 10% of the genes are shared among the top 200 genes (positive FC ranked, i.e genes upregulated in both populations) of each dataset. Whatever the strategy used by the authors to get to this final list of 3 genes, it needs to be more clearly explained and justified by a sound rationale. One would also expect to see the same type of analysis done with the “responding homogene cells” population, for comparison. If, as I would expect, more than 3 common genes are upregulated in these two populations of cells, it is difficult to hypothesize that “These genes … represent “cornerstones” of the trajectory of presumed gene expression changes towards new hair cells.”.
Other comments (pages not numbered on the pdf).
i) Introduction
- “These responding SC possess gene expression profiles similar to leukocytes” Where is this shown/formally analyzed?
- “Collectively, our results identify immune-like behavior in supporting cells followed by differentiation into nascent, regenerated hair cells.”. Implies too strongly that the “responding SC” are the ones (trans)differentiating into regenerated HC – not demonstrated conclusively, they could be clearing up debris or entering/triggering the mitotic regenerative response.
ii) Results
- “As expected, the lowest expressed genes are hair cell specific, many of which were recently validated by in situ hybridization (Janesick et al., 2021b).” Are all of the genes shown in the figure HC-specific? If not, highlight those that are.
- “We hypothesized that USP18 acts by suppressing the interferon response, allowing regenerated hair cell differentiation to commence. Furthermore, USP18 is expressed scarcely in ATOH1+ hair cells in the undamaged utricle (Scheibinger et al., 2021a), labelling new hair cells generated during natural turnover. Therefore, because it is linked to interferon genes, USP18 is likely specific to

© 2022. Published by The Company of Biologists under the terms of the Creative Commons Attribution License (https://creativecommons.org/licenses/by/4.0/).

responding supporting cells and regenerating hair cells and is not expressed when hair cells are naturally produced in the utricle without induced damage." I am confused by this section - clarify the "not expressed when hair cells are naturally produced…". Also, I could not find the result referred to in Scheibinger et al. 2021a.
- "At 48 hours post-sisomicin infusion, CALB2 was found in both medial and lateral responding supporting cells (CALB2/SOX2+), with a strong signal in the nuclei (Fig. 6F, F')." How is it known that these are indeed the "responding" supporting cells?
iii) Discussion
- "We found that JAK/STAT signaling was required because Ruxolitinib could effectively block expression of the damage induced interferon genes.". Cite Matsunaga et al. 2020 who reached a similar conclusion.
- Section "Regenerative Strategies Inferred at Three Weeks Post-Sisomicin Damage" distracts from the main focus of the study and given that the Discussion is already quite long, the authors might consider shortening this section.
iv) Figures
- several of the immunofluorescence figures would be difficult to visualize for a colour-blind reader. Check and adjust colour schemes when possible

---

First revision

Author response to reviewers' comments

Reviewer 1 Advance Summary and Potential Significance to Field...

The manuscript of Janesick et al. concerns the mechanisms that regulate the regeneration of sensory receptors in the avian inner ear. The manuscript has three primary foci, which are not entirely interrelated. First, the authors describe scRNA-Seq data of exceptionally high quality, demonstrating that numerous interferon signaling genes are expressed in response to hair cell injury. The second (somewhat related) focus is on the role of JAK/STAT signaling in regeneration. Using an epithelial culture system, it is demonstrated that inhibition of JAK leads to a reduction in regenerative proliferation, as well as decreased expression of interferon (INF) signaling genes. This part of the manuscript raises several questions that are not addressed. The third focus of this paper concerns the expression of calretinin (CALB2) is the regenerating basilar papilla. This issue is not explored in any detail and is not directly related to the other data reported in this manuscript.

Reviewer 1 Comments for the Author...

The manuscript of Janesick et al. has three primary foci, which are not entirely interrelated. First, the authors describe scRNA-Seq data of exceptionally high quality, demonstrating that numerous interferon signaling genes are expressed in response to hair cell injury. Those results are largely novel. Although (as acknowledged) some similar results have been reported by Matsunaga et al. (Front Neurosci, 2020), the present data are much more complete and thoroughly verified. As such, these data are a highly important contribution to the study of otic regeneration and implicate a role for inflammation (and inflammatory genes) in that process.

Response
We thank the reviewer for these positive comments about our manuscript. Our validations of the single cell RNA-seq analysis allowed us to pinpoint concretely which cell type (supporting cells) are up-regulating the interferon-response genes. We also show that infiltrating macrophages into the supporting cell layer are not responsible for the interferon response, which might have been presumed based on previous studies.
Response

The second (somewhat related) focus is on the role of JAK/STAT signaling in regeneration. Using an epithelial culture system, it is demonstrated that inhibition of JAK leads to a reduction in regenerative proliferation, as well as decreased expression of interferon (INF) signaling genes. This part of the manuscript raises several questions that are not addressed. First, what is the precise

© 2022. Published by The Company of Biologists under the terms of the Creative Commons Attribution License (https://creativecommons.org/licenses/by/4.0/).

relationship between JAK activation and INF signaling? It is commonly thought that JAK activation occurs downstream of INF activation (e.g., Mazewski et al., Front Immunol 2020). Are the authors suggesting that IFN triggers JAK/STAT, which then leads to expression of IFN signaling genes? The authors need to clarify this point.

Response
We are not suggesting that interferon ligand is the trigger. In our discussion, we explained that interferon receptors are lacking in the basilar papilla. Furthermore, other pattern- recognition receptors or immune sensors were not found to be expressed in the basilar papilla. One example, from our discussion is how intestinal epithelium mounts an interferon response after colonic injury that is required for regeneration and depends on EGFR and the ligand AREG (McElrath et al., 2021)...but not interferon ligands per se.

While we do believe that the response is mediated by JAK/STAT (due to our Rux inhibitor experiments), the trigger might not be interferon ligand. In fact, there are many categories of receptors linked to JAK/STAT intracellular signaling and likewise, a variety of ligands could be considered. At this point, the ligand/receptor that mediates JAK/STAT signaling during basilar papilla hair cell regeneration is unknown. Our ongoing research is focused on identifying the ligand and its receptor, but we argue that this project is a more substantial endeavor beyond the scope of the current paper. We have added additional clarifying statements in our discussion about this important point.
Response

Also (as acknowledged in the text), JAK can be activated by a number of upstream signals. I think that identifying those signal/signals in the damaged cochlea would be of critical importance.

Response
We agree that identification of the upstream signal/trigger is of critical importance but also very challenging to identify if #1. The signal might be coming from cells not characterized in this paper (e.g., non-epithelial cells) or #2. The signal becomes active from an existing pool or is released proteolytically and therefore is not detected via transcriptomic methods. #3 The ligand is very potent and mRNA levels are low. As we pointed out in our response above, we have added more clarification about this important challenge for research in this area.
Response

It is also possible that initial hair cell damage causes activation of IFN, which then causes activation of JAK/STAT, which then promotes expression of IFN target genes, thereby initiating a positive feedback loop that leads to further JAK activation, IFN gene expression, etc. It is notable that such a process would be blocked by RUX treatment that occurs shortly after injury.

Response
Yes, this is healthy conjecture, and we have added this to our discussion as a possible mechanism. While positive feedback loops do exist in biology, they are rare, and we are more intrigued by the negative feedback observed at 96-hours that we discussed in the paper. The chicken basilar papilla is mediating a strong response that is readily detectable by expression of interferon response genes, and moreover the tissue does an amazing job in shutting down the response before hair cell differentiation commences.
Response

To summarize: the demonstration that IFN signaling is activated after injury is a very interesting and important observation. More attention should be given to identifying the steps in that pathway that induced and regulate this activation.

Response
We completely agree and we are giving this subject more attention but also want to point out that it is the primary focus of future work. We hope that our more focused discussion of this point is satisfying the reviewer. It speaks to the importance of our observation that the immediate desire of a reader is to ask, “What ligand”? “What is the receptor”? Our intention is to provide the framework for these future research endeavors.
Response

© 2022. Published by The Company of Biologists under the terms of the Creative Commons Attribution License (https://creativecommons.org/licenses/by/4.0/).

Finally, what is the role of IFN gene expression in the process of regeneration? This presented data do not demonstrate that the downstream IFN genes are essential. It is conceivable that IFN signaling is activated by tissue damage, but that it plays NO role in triggering regeneration. If that were the case, the gene expression results described above would be of lesser interest. The presented data are very strong, but the authors need to clarify the causal link between IFN, JAK/STAT and hair cell regeneration.

Response
We absolutely agree with the reviewer that identifying the causal link between a trigger and subsequent hair cell regeneration is a holy grail. We have identified a piece of the story by showing that interferon response genes are activated during regeneration in supporting cells and we show that this activation is mediated via JAK/STAT signaling. The data presented in our paper do not establish a definitive, causal link between the interferon response and hair cell regeneration. We humbly assert that if this were the burden of proof required for publication, then having such data in hand would essentially solve the mechanism of chicken hair cell regeneration (a 30+ year old mystery). To say it is of lesser interest is to ignore the reality that complete understanding of chicken hair cell regeneration will likely not occur suddenly with one definitive paper, but rather with individual contributions, and smaller building blocks to get there. This said, we revised our discussion to place our observation and validation experiments into the context of the bigger picture questions.
Response

The authors should also provide some rationale for use of the epithelial culture system. It could be argued that isolated epithelia, adhering to a glass substrate via Cell Tak, are not an optimal model for these experiments. The native environment (e.g., ECM proteins, etc.) provided by organotypic cultures may be more realistic. I am not requesting that the authors repeat these studies, but the pro’s and con’s of the epithelial culture system should be discussed.

Response
We added a rationale for using the method we chose. We indeed observed the same results (strong up-regulation of interferon genes, followed by the inhibition of interferon response with RUX) when using other substrates like Matrigel. We chose Cell Tak because it was the best substrate for keeping the epithelium adhered. Free-floating peeled epithelium tends to get more mangled during culture and it was not easy to maintain the tissue’s structural organization in these experiments.

It should be noted, though, that we observed the same interferon response when we cultured whole basilar papillae. The fold changes, however, were lower since there were many other cell types contributing to the pool of RNA harvested for the qPCR analysis.
Therefore, we chose to show data from what we believe to be a cleaner experiment.

Furthermore, peeled epithelia cultures are more comparable and consistent with the single cell data (which is also peeled epithelia only).

We have added statements regarding our culturing method of choice to the revised manuscript. We thank the reviewer for pointing out the different techniques which all present their own limitations. Whole cochlear ducts are heterogeneous and therefore bulk qRT-PCR or other bulk analysis methods might produce more diluted measurements compared to peeled tissue where the protagonists are more enriched, but not surrounded by a more native environment. Of course, whole cochlear ducts, followed by single-cell analysis methods (at various time points) would be a very efficient way to pursue this challenge. This is certainly a research direction we will pursue in future studies – likely utilizing our in vivo model system.
Response

The third focus of this paper concerns the expression of calretinin (CALB2) is the regenerating basilar papilla. The authors show expression of CALB2 in both regenerative supporting cells as well as in replacement hair cells. The supporting cell expression is novel and likely to be interesting. However, this issue is not explored in any detail and is not directly related to the other data reported in this manuscript.

© 2022. Published by The Company of Biologists under the terms of the Creative Commons Attribution License (https://creativecommons.org/licenses/by/4.0/).

Response
We validated the single cell transcriptomic expression of CALB2 with immunohistochemistry and showed co-expression of CALB2 protein with proliferating EdU+ cells in Figure 6F, F'.
CALB2 expression in proliferating supporting cells shows that the upregulation of this gene coincides with the interferon response which we show with sections from the 48-hour timepoint that feature expression of IFI6 and CALB2 in regenerative supporting cells (please compare Figures 3D and Figure 6D for co-expression in responding supporting cells; in addition to Figure 6F, F'). We feel that this experimental evidence, in addition to the single- cell RNA-seq results, provides solid validation of our observation.
Response

I recommend omitting those data from the present paper and publishing them once a more complete story has been obtained. The demonstration of inflammatory signaling is of great interest and should really be the focus of this paper.

Response
We would prefer not to omit the CALB2 data. The presence of CALB2 in both proliferating supporting cells and new hair cells is a novel finding that should be shared with the scientific community. Follow-up experiments surrounding CALB2 function would probably best be conducted by those with strong Ca^{2+} physiology expertise to understand how calcium sensoring and buffering might contribute to hair cell regeneration. Since such experimentation would be outside our expertise, we much prefer to share this result here with the broader scientific community, rather than hold back the result for follow-up experiments we are likely to never pursue ourselves.
Response

In summary, the authors have performed an outstanding genomic characterization of the regenerating chick cochlea, which resulted in the very intriguing suggestion that IFN signaling may be involved in the regenerative process. The study requires a more mechanistic picture of the role of IFN signaling in the regenerative process.

Minor issues:
(1) The authors appear confident about the purity of their cell isolation system, i.e., that it yields only cells from the sensory epithelium of the basilar papilla. If this is true, then we are there small clusters of red and white blood cells in the tSNE plots shown in Fig. 2?

Response
The clusters of blood cells in the tSNE plots represent a very small fraction of the total number of cells collected. Additional purity could be achieved by using flow cytometry, which would lead to other limitations. Because blood cells are easily identified bioinformatically, we did not perceive this as a problem. We added some words in the methods section that puts our statement about purity into context.
Response

(2) Does hair cell production in the superior region of the papilla occur via symmetric or asymmetric division (or both)? If it occurred vis asymmetric proliferation, then we would expect to see approximately equal numbers of labeled hair cells and supporting cells. In fact, they differ (87% vs. 59%).

Response
The consensus in the field is that hair cell production in the superior region of the BP occurs by asymmetric division where a supporting cell divides and becomes one supporting cell and one hair cell. The explanation for why 59% of medial supporting cells incorporate EdU while 87% of hair cells incorporate EdU is simple…not all supporting cells proliferate. There are ~1.8x more supporting cells than hair cells on the medial side (Janesick and Heller, 2019), so not every supporting cell needs to engage in proliferation to replenish the hair cells. To this point, a larger single cell experiment (collecting many more cells than the present study) might show two superior supporting cell populations (one that is cycling, and one that is not). We did not have the resources to conduct the single-cell analysis at such a refined resolution to see this in the present study.
Response

© 2022. Published by The Company of Biologists under the terms of the Creative Commons Attribution License (https://creativecommons.org/licenses/by/4.0/).

(3) The discussion of phalangeal scaring in the cochlea is somewhat misleading. The data in the cited paper (Lee et al., Neurosci 2021) do not show clear evidence for MET in the damaged cochlea. Instead, that study characterized the process of epithelial repair (via rearrangement at the lumen), and also that Deiters cells could expand and engulf prestin- labeled debris of dead OHC's. The results of Hu and Corwin (2007) are derived from cultures of isolated supporting cells and their relevance to repair in vivo are not clear. In any case, the use of the term 'scar' to describe this process is unfortunate, because it implies certain similarities between epithelial repair in the inner ear and epidermal wound healing. Epithelial repair in the ear is (usually) quite simple, i.e., adjoining supporting cells expand to reseal gaps in the epithelial barrier caused by the death of hair cells. Some supporting cells may also engulf cellular debris. However, except in extreme situations, repair to the cochlea does not involve EMT, MET or fibrosis. I acknowledge that the use of the term 'scar' to describe cochlear repair has been common for ~30 years and certainly did not originate with the present authors. But, because that term is applied to repair in the cochlea, it is not fair to assume that other features of tissue scarring in (for example) skin wounds also occur in the ear.

Response
We agree that these statements were indeed misleading. Because this specific discussion did not add substantial depth to our discussion of the results, we have removed the section.
Response

(4) In the Discussion, the author quote H.W. Longfellow, but no citation is given for this quotation. Such omission could be detrimental to Mr. Longfellow's h-index and diminish the significance of his literary career.

Response
Very funny....The citation is now provided.
Response

Reviewer 2 Advance Summary and Potential Significance to Field...

This paper is the first to use single-cell RNA-Seq to analyse gene expression levels in the course of hair cell (HC) regeneration in the chicken basilar papilla (BP). This organ is an important model system for HC regeneration studies: following HC death, the supporting cells that surround them can give rise to new HC by direct transdifferentiation or by a mitotic route. The signals that initiate the regenerative response are unknown and transcriptome analyses such as those performed in this study could potentially shed new light on their nature.

Here, the authors use the intra inner ear injection of an aminoglysocide antibiotic to induce rapid, massive and synchronous HC death throughout the BP of young chicks. This protocol has been described by the authors in a recent study investigating the changes in gene expression during hair cell death in the BP. This study is the natural extension of this work, focusing on genes expressed within the regenerating epithelium in the days following hair cell death.

Their findings from bulk and single-cell RNA-seq datasets collected and analysed at different time points post-damage confirm recent work (Matsunaga et al. 2020) and show that i) the expression of several interferon response genes is upregulated in a subset of "responding" supporting cells (the progenitors for new hair cells) after hair cell damage and that ii) the JAK/STAT pathway is required for this response. The single-cell analyses reveal different clusters of supporting cells post-damage, which could represent cells engaging in different types of regenerative/proliferative responses; a new marker for regenerated hair cells, calretinin (CALB2), is also identified.

Finally, the paper describes the spatio-temporal pattern of hair cell regeneration and supporting cell proliferation in their ototoxicity model.

The main advance provided by this study is the single-cell RNA-Seq dataset itself, which represents a very valuable resource for the inner ear research community: the experiments are well designed and executed, several time points post-damage are analysed, bulk versus single-cell RNA-Seq datasets are compared, and the bioinformatics analyses suggest high- quality, information-rich datasets.

© 2022. Published by The Company of Biologists under the terms of the Creative Commons Attribution License (https://creativecommons.org/licenses/by/4.0/).

On the other hand, the actual role of interferon signalling in the context of hair cell regeneration remains unclear, as is the potential role of the other genes singled-out by the authors in their bioinformatics analyses. The conceptual advances, as far as the molecular basis/triggers of hair cell regeneration is concerned, are therefore limited. Furthermore, the rationale and interpretation of some of the analyses trying to establish the “link” between responding supporting cells and the newly regenerated hair cells are unclear to me.

Response
We thank the reviewer for the encouraging summary. We agree that the single cell data will provide a rich resource to the inner ear field and we have created a gEAR profile that will allow users to easily access the data for their own needs.

We acknowledge the reviewer’s point about conceptual advances. Please also see our responses to Reviewer 1 about this point in general. Yes, our study did not reveal the specific ligand/receptor complex that ultimately triggers upregulation of interferon response genes. Likewise, we were did not definitively link between interferon response gene upregulation and S-phase entry of supporting cells. On the other hand, we have identified and unequivocally validated a novel pathway that is intriguingly active in regenerating supporting cells. Moreover, our functional analyses with pathway inhibitors provide confidence that JAK/STAT signaling is active in regenerating supporting cells and that there is a strong gene regulatory response. We feel that this is an important result that, of course, raises many additional questions. Our data therefore provides clues towards many follow up experiments that - we hope - will ultimately provide a basis for unraveling the signaling events that control mitotic hair cell regeneration in the chicken basilar papilla.
Response

Reviewer 2 Comments for the Author...

Main comments:
- Role of JAK/STAT in HC regeneration. There is now strong evidence, based on the present study and the recent one by Matsunaga et al (2020) that interferon-response genes are upregulated in SC following HC death. The pharmacological inhibition of JAK/STAT activity with RUX in vitro (also previously performed by Matsunaga et al 2020) confirm the implication of this pathway in the upregulation of interferon-response genes in SC, but one wonders why the authors did not investigate the effects of RUX on hair cell regeneration itself. This would have allowed much stronger conclusions to be drawn from the study.

Response
Hair cell regeneration in the culture system, while occasionally performed in the field, is incomplete. Regenerating hair cells in culture (either peeled or whole BP) was never robust enough in our hands to firmly quantitate the number of Edu+ cells and new hair cells observed +/− Rux inhibitor. Such challenges were the impetus to develop the surgical model in the first place. And while we are proud of the technological advance we developed to get aminoglycosides into the chicken ear, there is still progress to be made for delivery of other drugs. This is a subject of ongoing research for everyone in the field…how to get viruses or therapeutics into the ear…the successful delivery is based on numerous factors (Plontke and Salt, 2018).

We indeed attempted to inject Rux both subcutaneously (as we do EdU injections) and via canalostomy, and did not observe that hair cell regeneration was blocked. We were hesitant to add these data in the paper, not because they are negative results, but because they are inconclusive. For example, we were unclear whether Rux was successfully reaching sufficient concentration and a prolonged presence in the inner ear.

We are currently testing in vivo infusion of various water soluble, small-molecule inhibitors which target various pathways of interest, including the interferon pathway. These results will be published in subsequent studies. We would like to note that these experiments are being conducted in vivo for obtaining the most significant results and are therefore extensive and time-consuming.
Response

© 2022. Published by The Company of Biologists under the terms of the Creative Commons Attribution License (https://creativecommons.org/licenses/by/4.0/).

I was also surprised to see in Fig. 5A that RUX induces a significant upregulation of the expression of HC-specific genes in control cultures; this result is not mentioned in the text of the legend/results, but it suggests to me that RUX could potentially impact on cell composition/transcription in the absence of damage. One can appreciate that a comprehensive study of the effects of RUX on HC regeneration may be beyond the scope of this study, but it seems that a simple immunostaining for HC/SC markers at the end of the RUX treatment in vitro could have helped to resolve some of these questions.

Response
Thank you for bringing this to our attention. For this replicate we also observed slightly increased CCL4 and TMSB4X expression, suggesting that perhaps something is slightly off with housekeeping normalization. But, since we do not observe elevated expression across ALL genes tested, we cannot exclude this data as an outlier. There are other tiny quirks in the data, like elevated TECTB and OTOGL expression in the SISO+RUX condition. A simple immunostaining for HC/SC is not necessarily going to resolve this question. We do not have antibodies for SLC34A2 or TMEM255B but could use other tools. Chasing down these results (often driven by one replicate) are challenging. We added a note to the figure legend saying that upregulation of HC and SC genes in response to RUX was observed in isolated experiments but was neither consistent nor significant across all replicates.
Response

- Section "CALB2, USP18, and TRIM25 link responding supporting cells to new hair cells". Early in this section, the authors write that: "Our next goal was to link the responding supporting cell population to new hair cells.", which suggests a strong bias in their exploration of the data… the analyses themselves reinforce this sentiment and do not, in my view, support the conclusions of the authors:

Response
We have deleted this statement.
Response

"In lieu of a trajectory analysis, we compared the top-ranking genes of the new hair cell and the responding supporting cell group and found three genes present in both: CALB2, USP18, and TRIM25 (Fig. 6A-D)." What exactly are these comparisons? Are the top-ranking genes in these two datasets selected according to log2FC or FDR values? How many top-ranking genes from each dataset are compared? From a rapid survey of the supplementary files for the DEG in "responding SC" and "new HC", I found that about 10% of the genes are shared among the top 200 genes (positive FC ranked, i.e genes upregulated in both populations) of each dataset. Whatever the strategy used by the authors to get to this final list of 3 genes, it needs to be more clearly explained and justified by a sound rationale.

Response
We eagerly provide more explanation in the methods and apologize for the omission. Yes, the top-ranking genes from both groups (responding supporting cells and new hair cells) were compared. We defined "top-ranking" using the standard single cell thresholding convention mentioned in this paper, and also throughout our baseline paper (Janesick et al., 2021): FDR < 0.01 and log2FC > 2. Many single-cell differential gene expression analyses use expression differences > 2-fold; we use > 4-fold. We realize that this threshold might seem stringent, but it is based on validation results from our baseline paper. Using this threshold, we can confidently validate nearly 100% of genes by in situ hybridization. A less stringent threshold results in decreasing validation robustness.

If you run the "survey" with these metrics, you will find CALB2 and USP18. TRIM25 is borderline (and you can see from the tSNE in Figure 6C, its expression is not as clear-cut as CALB2 and USP18), and we are happy to remove this gene from our discussion, but we were tempted to include it based on its connection to interferon signaling.

We did find a typo in Figure 6A, which might have caused some confusion for the reviewer as the label for "responding SCs" and "new HCs" were accidentally swapped. Indeed SCs and responding SCs are much more similar to each other than new and mature HCs, which have many more

© 2022. Published by The Company of Biologists under the terms of the Creative Commons Attribution License (https://creativecommons.org/licenses/by/4.0/).

differentially expressed genes between them. The new HC circle in the Venn diagram should obviously be the larger one. This has now been corrected.
Response

One would also expect to see the same type of analysis done with the “responding homogene cells” population, for comparison. If, as I would expect, more than 3 common genes are upregulated in these two populations of cells, it is difficult to hypothesize that “These genes … represent “cornerstones” of the trajectory of presumed gene expression changes towards new hair cells.”.

Response
We ran this same analysis in responding homogene cells and new hair cells and found that USP18 indeed is present in both groups, but not CALB2 or TRIM25. We are de-prioritizing a trajectory between responding homogene cells and hair cells…this doesn’t make our analysis biased, rather, we are following the biology where it is the supporting cells that regenerate hair cells, not homogene cells. All data is available for individual analyses at gEAR and we hope that our paper is seen as a resource that inspires readers to conduct their own comparative analyses, driven by their individual interests. We added a statement along these lines to the revised text.
Response

Other comments (pages not numbered on the pdf).

Response
For the resubmission we have inserted line numbers and apologize for their omission in the original submission.
Response

i) Introduction
- “These responding SC possess gene expression profiles similar to leukocytes” Where is this shown/formally analyzed?

Response
This is best found in the gene ontology analysis (Figure 1C). The Reactome database used in our analysis is agnostic to cell types. Enrichr, in contrast, provides cell atlasing, and here are the results (using top-ranking responding supporting cell genes):

HuBMAP Consortium: Cd4 T Cells, T agonist, T Follicular Helper Cell
CCLE Proteomics 2020: H1650 -- a lung cancer cell line with EGFR mutation CCLE Proteomics 2020: PL45 -- pancreatic adenocarcinoma epithelial cell line Cell Marker Augmented 2021: Exhausted CD8+ T cell (Liver)
PanglaoDB 2021: Trophoblast progenitor cells, microfold cells (lymphoid), monocytes Azimuth Cell Types 2021: CD16+ Monocytes
BioGPS Mouse Gene Atlas: macrophage peri LPS thio 7hrs
ARCHS4 Tissues: CD34+ cell, plasmacytoid dendritic cell, bone marrow, cord blood
Response

- “Collectively, our results identify immune-like behavior in supporting cells followed by differentiation into nascent, regenerated hair cells.”. Implies too strongly that the “responding SC” are the ones (trans)differentiating into regenerated HC – not demonstrated conclusively, they could be clearing up debris or entering/triggering the mitotic regenerative response.

Response
We have rephrased the sentence and removed the reference to “immune-like”.
Perhaps not conclusively demonstrated in the single cell data without more timepoints collected. However, IFI6, IFIT5, and OASL are expressed quite ubiquitously (not sparsely) in the medial supporting cells (see Figure 3D, E, F). And we know these cells proliferate and differentiate into hair cells. We have conducted additional experiments to conclusively show that EdU incorporation happens in IFI6 expressing supporting cells at 48 hours post- sisomicin. This is shown below.

© 2022. Published by The Company of Biologists under the terms of the Creative Commons Attribution License (https://creativecommons.org/licenses/by/4.0/).

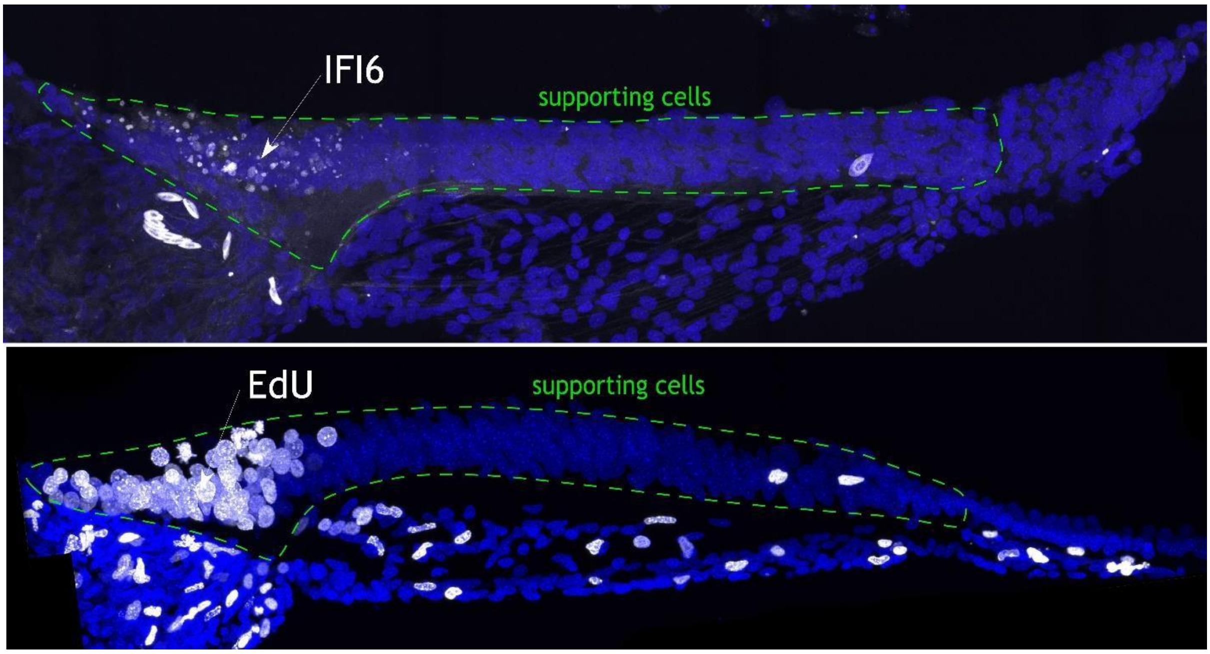

Response

ii) Results

- "As expected, the lowest expressed genes are hair cell specific, many of which were recently validated by in situ hybridization (Janesick et al., 2021b)." Are all of the genes shown in the figure HC-specific? If not, highlight those that are.

Response

In Figure 1B, all genes are hair cell specific except A2M and COL14A1 (supporting cell markers), and CALB1 (which is both hair cell and supporting cell marker). We have revised the text to reflect this.

Response

- "We hypothesized that USP18 acts by suppressing the interferon response, allowing regenerated hair cell differentiation to commence. Furthermore, USP18 is expressed scarcely in ATOH1+ hair cells in the undamaged utricle (Scheibinger et al., 2021a), labelling new hair cells generated during natural turnover. Therefore, because it is linked to interferon genes, USP18 is likely specific to responding supporting cells and regenerating hair cells and is not expressed when hair cells are naturally produced in the utricle without induced damage." I am confused by this section – clarify the "not expressed when hair cells are naturally produced…". Also, I could not find the result referred to in Scheibinger et al.
2021a.

Response

Sorry for the confusion. In the utricle, hair cells undergo a natural turnover process, which is a form of regeneration, but not injury-induced. When we revisited these data from Scheibinger et al. 2021a we found that USP18 is expressed to some degree in a small group of Atoh1+ cells of the utricle. Therefore, in the manuscript text, we deleted any statement regarding USP18 and the utricle. We are working to make these data more accessible and a gEAR profile that accompanies Scheibinger et al. 2021a is available here: https://umgear.org/index.html?layout_id=3522046d

Response

- "At 48 hours post-sisomicin infusion, CALB2 was found in both medial and lateral responding supporting cells (CALB2/SOX2+), with a strong signal in the nuclei (Fig. 6F, F')." How is it known that these are indeed the "responding" supporting cells?

© 2022. Published by The Company of Biologists under the terms of the Creative Commons Attribution License (https://creativecommons.org/licenses/by/4.0/).

Response
In this same figure, we show co-staining of CALB2 with EdU, so these cells are actively proliferating. EdU incorporation never occurs in the quiescent, undamaged cochlea, which is why we conclude that these supporting cells are responding.
Response

iii) Discussion
- "We found that JAK/STAT signaling was required because Ruxolitinib could effectively block expression of the damage induced interferon genes.". Cite Matsunaga et al. 2020 who reached a similar conclusion.

Response
Cited.
Response

- Section "Regenerative Strategies Inferred at Three Weeks Post-Sisomicin Damage" distracts from the main focus of the study and given that the Discussion is already quite long, the authors might consider shortening this section.

Response
We have removed the section on scarring, as requested by the other reviewer which should make the discussion shorter.
Response

iv) Figures
- several of the immunofluorescence figures would be difficult to visualize for a colour-blind reader. Check and adjust colour schemes when possible

Response
We have adjusted Figure 4 to display hair cells in yellow, dead hair cell corpses in magenta, and tinted the green supporting cells more towards a cyan hue. We adjusted Figure 6 to display EdU+ cells in magenta and S4 to display dead hair cells in magenta.
Response

Second decision letter

MS ID#: DEVELOP/2021/200113

MS TITLE: Chicken Auditory Supporting Cells Express Interferon Response Genes during Regeneration towards Nascent Sensory Hair Cells In Vivo

AUTHORS: Amanda S Janesick, Mirko Scheibinger, Nesrine Benkafadar, Sakin Kirti, and Stefan Heller

I have now received all the referees reports on the above manuscript, and have reached a decision. The referees' comments are appended below, or you can access them online: please go to BenchPress and click on the 'Manuscripts with Decisions' queue in the Author Area.

The overall evaluation is positive and we would like to publish a revised manuscript in Development, provided that the referees' comments can be satisfactorily rebutted; you will see that one of the reviewers still has significant concerns that we would like you to address. Please attend to all of the reviewers' comments in your revised manuscript and detail them in your point-by-point response. If you do not agree with any of their criticisms or suggestions explain clearly why this is so.

We are aware that you may currently be unable to access the lab to undertake experimental revisions. If it would be helpful, we encourage you to contact us to discuss your revision in greater detail. Please send us a point-by-point response indicating where you are able to address concerns

© 2022. Published by The Company of Biologists under the terms of the Creative Commons Attribution License (https://creativecommons.org/licenses/by/4.0/).

raised (either experimentally or by changes to the text) and where you will not be able to do so within the normal timeframe of a revision. We will then provide further guidance. Please also note that we are happy to extend revision timeframes as necessary.

Reviewer 1

Advance summary and potential significance to field

As noted in the prior reviews, this manuscript contains data of very high quality, characterizing gene expression in the chick cochlea after injury and during regeneration. Data consist of both bulk and single cell RNA-Seq, with excellent in situ verification. A key finding is that interferon (IFN) signaling is activated in response to hair cell injury. However, IFN ligands and receptors are not present, so IFN activation much occur through a noncanonical mechanism.
Expression of IFN signaling genes is reduced by small molecule inhibition of JAK/STAT signaling, but there is no evidence that the JAK/STAT pathway is activated in this situation. Finally, it is suggested that IFN signaling is involved in regeneration, but no direct evidence is provided.

Comments for the author

Although the data are exceptional, there remain numerous unresolved issues. As a result, it is difficult establish any clear chain of causality from the reported findings. Resolution of (at least) some of the following points would greatly strengthen this manuscript.
1. The bulk RNA-Seq data clearly show enhance abundance of interferon-associated genes (Fig. 1). This prompted efforts to identify those cells, using scRNA-Seq methods (Figs. 2,3). Bulk RNA-Seq data depends on the total reads for each gene, and a high ranking could indicate that a gene is expressed by most or all cells (but at rather moderate levels) or is expressed at high levels by a subset of cells. The data for IFN genes appear more consistent with the second category, i.e., they are expressed only in the superior-most supporting cells and in some homogene cells. Notably, the superior-most supporting cells are also the cells that proliferate after hair cell injury. This is a provocative finding, but the association between interferon signaling and regeneration is largely circumstantial.
2. Clearly, if IFN signaling is involved in regeneration, it only serves this role in a limited portion of the sensory epithelium. The spatial restriction of IFN gene expression should be emphasized. I also recommend that such supporting cells be labeled as “IFN-responding” (or something similar), rather than “responding.” Because regeneration occurs across the sensory epithelium, it is reasonable to assume that the inferior/abneural supporting cells are also “responding” to hair cell loss, even though they did not respond by expressing IFN genes.
3. It is curious that the region that expresses IFN response genes after injury corresponds with the regions in which the chemokine CLCX14 is expressed in hair cells. Injury to hair cells might either disrupt ongoing CXCL14 signaling (between hair cells and supporting cells) or may result in extracellular release of CXCL14. Is it possible that either of these processes may activate IFN signaling?
4. In the manuscript, it is proposed that production of interferon (IFN) in response to hair cell injury is responsible for JAK/STAT activation and subsequent regeneration in the cochlea. In their response to the initial reviews, the authors acknowledge that receptors for IFN are not expressed in the cochlea. Instead, they propose a noncanonical IFN response that has been shown to occur in the injured gut epithelium (McElrath et al., 2021). Unfortunately no supporting data are provided.
5. The authors test the role of JAK/STAT signaling in regeneration by blocking its activation with RUX. Curiously, they do not show direct evidence that JAK/STAT is actually activated by hair cell injury. Given the link between JAK/STAT and IFN genes, we might expect JAK/STAT to be activated only in the superior region of the injured sensory epithelium. Are there good histological markers for JAK/STAT activation (e.g., antibodies, etc.)? Can scRNA-Seq data be used to show evidence for JAK/STAT activation in the ‘responding’ supporting cells? Or is the expression of IFN response genes sufficient evidence for activation of JAK/STAT?
6. The authors also acknowledge that JAK/STAT signaling might be activated by other upstream signals (rather than IFN). Still, no evidence for the identity of these signals is given. It is stated that such signals would be “very challenging to identify.” I agree with this assessment and certainly sympathize with the author’s predicament. However, with the current data, it is impossible to make a firm correlation between IFN signaling, JAK/STAT activation and subsequent regeneration.

© 2022. Published by The Company of Biologists under the terms of the Creative Commons Attribution License (https://creativecommons.org/licenses/by/4.0/).

7. The fact RUX treatment reduced expression of all IFN response genes except CCL4 suggests that this chemokine may be serving a different function than the other genes. One plausible role for CCL4 is a macrophage chemoattractant.
8. Both of the previous reviews noted that the data concerning CALB2 were quite distinct from the issues discussed above and, as a result, the manuscript appears to be unfocused. This is still the case.
In summary, I recommend that the authors provide some direct evidence of JAK/STAT activation and further show that the pathway is blocked by RUX.

<u>Reviewer 2</u>

Advance summary and potential significance to field

I am satisfied with the answers to my queries/comments and the revisions to the manuscript. My main criticism was about the way the "link" between "responding SC" and "new HCs" was established/discussed and I thank the authors for clarifying their methodology. There is still room for debate about the significance of this link, and how are the "responding SC" contributing to the regenerating response... but overall the study provides extremely valuable insights into the molecular correlates of hair cell regeneration and I am looking forward to seeing the results of follow-up experiments on the specific roles of interferon signalling.

Three minor points:
1) I think it is worth reminding the reader about the definition of "top ranking" genes (FDR<0.01 and Log2FC>2) in the section about common genes between responding SCs and new HCs (line 254). It was not clear to me that this was a standard for this paper, and in fact other sections rely on different thresholds (see line 104/Figure 1).
2)The "*" representing statistical significance are now largely absent from Figure 5?
3) In Figure 6F' it remains hard to see, at the individual cell nucleus level, the proportion of calretinin-positive cells that are also Edu+ (Sox2 signal is not helping). There's certainly some spatial overlap on the medial side, but not a strict correlation at the individual cell level. In fact, only a subpopulation of the calretinin+/Sox2+ SC are Edu+.. You could add a high mag view of samples stained for Edu/calretinin only to make this clearer and/or rephrase the statement line 265-66 (eg "...these supporting cells were in the proliferative phase of regeneration" changed to "... at least some of these supporting cells were in the proliferative phase...").

Comments for the author

I am satisfied with the answers to my queries/comments and the revisions to the manuscript. My main criticism was about the way the "link" between "responding SC" and "new HCs" was established/discussed and I thank the authors for clarifying their methodology. There is still room for debate about the significance of this link, and how are the "responding SC" contributing to the regenerating response... but overall the study provides extremely valuable insights into the molecular correlates of hair cell regeneration and I am looking forward to seeing the results of follow-up experiments on the specific roles of interferon signalling.

Three minor points:
1) I think it is worth reminding the reader about the definition of "top ranking" genes (FDR<0.01 and Log2FC>2) in the section about common genes between responding SCs and new HCs (line 254). It was not clear to me that this was a standard for this paper, and in fact other sections rely on different thresholds (see line 104/Figure 1).
2)The "*" representing statistical significance are now largely absent from Figure 5?
3) In Figure 6F' it remains hard to see, at the individual cell nucleus level, the proportion of calretinin-positive cells that are also Edu+ (Sox2 signal is not helping). There's certainly some spatial overlap on the medial side, but not a strict correlation at the individual cell level. In fact, only a subpopulation of the calretinin+/Sox2+ SC are Edu+.. You could add a high mag view of samples stained for Edu/calretinin only to make this clearer and/or rephrase the statement line 265-66 (eg "...these supporting cells were in the proliferative phase of regeneration" changed to "... at least some of these supporting cells were in the proliferative phase...").

© 2022. Published by The Company of Biologists under the terms of the Creative Commons Attribution License (https://creativecommons.org/licenses/by/4.0/).

Second revision

Author response to reviewers' comments

Reviewer 1 Advance Summary and Potential Significance to Field...

As noted in the prior reviews, this manuscript contains data of very high quality, characterizing gene expression in the chick cochlea after injury and during regeneration. Data consist of both bulk and single cell RNA-Seq, with excellent in situ verification. A key finding is that interferon (IFN) signaling is activated in response to hair cell injury. However, IFN ligands and receptors are not present, so IFN activation much occur through a noncanonical mechanism. Expression of IFN signaling genes is reduced by small molecule inhibition of JAK/STAT signaling, but there is no evidence that the JAK/STAT pathway is activated in this situation. Finally, it is suggested that IFN signaling is involved in regeneration, but no direct evidence is provided.

We identify a group of genes named “interferon-induced genes,” such as IFIT5 and IFI6. The reviewer misunderstood that this means that we propose that interferon (IFN) signaling is activated in response to hair cell loss. Gene nomenclature is sometimes misleading. IFI and IFIT genes are upregulated via JAK/STAT signaling, which is linked to many receptors and ligands. To be clear: IFI and IFIT genes are upregulated by many different cytokines and not solely by IFNs. Therefore, it would be an over- simplification to say that activation occurs via a noncanonical mechanism.

We checked and changed the text in this second revision to avoid linking IFN signaling to the reported damage response. We also added a statement that clarifies the unfortunate existinomenclature “interferon response genes” to point out that many signaling pathways regulate these genes, including various cytokines and not necessarily interferons. We also acknowledge that a limitation of our study is that we cannot point to a specific cytokine responsible for the activation of the JAK/STAT signaling pathway in supporting cells.

Moreover, we removed any direct statements about IFN signaling and possible relation to hair cell regeneration, and replaced “interferon response genes” simply with “immune-related genes” or “JAK/STAT signaling response genes.”

We also changed the title of the manuscript to account for these edits.

Finally, we avoided concluding remarks directly linking IFN and JAK-STAT signaling to hair cell regeneration. Nevertheless, we kept interpretative statements such that our findings “suggest a potential functional involvement of JAK/STAT signaling and responding genes in hair cell regeneration.”

We hope these clarifications and manuscript revisions provide a satisfactory response to this critique.

Reviewer 1 Comments for the Author...

Although the data are exceptional, there remain numerous unresolved issues. As a result, it is difficult establish any clear chain of causality from the reported findings. Resolution of (at least) some of the following points would greatly strengthen this manuscript.

We thank the reviewer for the positive words about are data. Please see our responses below.

1. The bulk RNA-Seq data clearly show enhance abundance of interferon-associated genes (Fig. 1). This prompted efforts to identify those cells, using scRNA-Seq methods (Figs. 2,3). Bulk RNA-Seq data depends on the total reads for each gene, and a high ranking could indicate that a gene is expressed by most or all cells (but at rather moderate levels) or is expressed at high levels by a subset of cells. The data for IFN genes appear more consistent with the second category, i.e., they are expressed only in the superior-most supporting cells and in some homogene cells. Notably, the

© 2022. Published by The Company of Biologists under the terms of the Creative Commons Attribution License (https://creativecommons.org/licenses/by/4.0/).

superior-most supporting cells are also the cells that proliferate after hair cell injury. This is a provocative finding, but the association between interferon signaling and regeneration is largely circumstantial.

We have clarified in the text that the immune-related gene expression in the bulk RNA-seq data is likely caused by a subset of supporting cells. Also, we acknowledged that our in situ hybridization revealed medial expression of genes, and added text to support this.

2. Clearly, if IFN signaling is involved in regeneration, it only serves this role in a limited portion of the sensory epithelium. The spatial restriction of IFN gene expression should be emphasized. I also recommend that such supporting cells be labeled as "IFN-responding" (or something similar), rather than "responding." Because regeneration occurs across the sensory epithelium, it is reasonable to assume that the inferior/abneural supporting cells are also "responding" to hair cell loss, even though they did not respond by expressing IFN genes.

Please see our responses above and the edits in the manuscript to avoid confusion about a role for interferons. We feel that removing the term "interferon-induced genes" and revising our statements that the observed robust upregulation of immune-related genes is linked to JAK/STAT signaling now provides more clarity.

We added text in lines 228-230 to emphasize the point about the spatial restriction of IFN gene expression. Based on our reasoning, we would like to avoid calling supporting cells "IFN-responding" because this might suggest that these cells respond to IFN ligand, which we have not proven to be the trigger.

3. It is curious that the region that expresses IFN response genes after injury corresponds with the regions in which the chemokine CLCX14 is expressed in hair cells. Injury to hair cells might either disrupt ongoing CXCL14 signaling (between hair cells and supporting cells) or may result in extracellular release of CXCL14. Is it possible that either of these processes may activate IFN signaling?

Discussing a potential role for CXCL14 is speculative at this point because we found no conclusive evidence for CXCL14 signaling between hair cells and supporting cells. CXCL14 is one of the few CXC- ligands for which a receptor has not been identified, which makes a discussion of concrete involvement challenging.

4. In the manuscript, it is proposed that production of interferon (IFN) in response to hair cell injury is responsible for JAK/STAT activation and subsequent regeneration in the cochlea. In their response to the initial reviews, the authors acknowledge that receptors for IFN are not expressed in the cochlea. Instead, they propose a noncanonical IFN response that has been shown to occur in the injured gut epithelium (McElrath et al., 2021). Unfortunately, no supporting data are provided.

Indeed, we found no IFN receptors expressed in the cochlea. Likewise, there is no expression of IFN ligands detectable. In the discussion, we introduce references for induction of immune-related genes by cytokines, such as McElrath et al. We feel that clarification that the upregulated genes require JAK/STAT signaling and removal of the statements specifically linking IFN signaling provides a less speculative manuscript.

5. The authors test the role of JAK/STAT signaling in regeneration by blocking its activation with RUX. Curiously, they do not show direct evidence that JAK/STAT is actually activated by hair cell injury. Given the link between JAK/STAT and IFN genes, we might expect JAK/STAT to be activated only in the superior region of the injured sensory epithelium. Are there good histological markers for JAK/STAT activation (e.g., antibodies, etc.)? Can scRNA-Seq data be used to show evidence for JAK/STAT activation in the 'responding' supporting cells? Or is the expression of IFN response genes sufficient evidence for activation of JAK/STAT?

We show that the upregulation of immune-related genes is inhibited in presence of a blocker of JAK/STAT signaling (RUX). We revised our statements to focus on this specific point which we can support with data.

© 2022. Published by The Company of Biologists under the terms of the Creative Commons Attribution License (https://creativecommons.org/licenses/by/4.0/).

We have tested STAT1 and STAT3 antibodies in tissue, but these antibodies (which are designed to mammalian epitopes) do not appear to work in immunohistochemistry in chickens. We have preliminary evidence that STAT3 is up-regulated using Western blots, but this experiment does not preserve the spatial information of the tissue.

STAT1 is significantly up-regulated in supporting cells when we assess the scRNA-seq data, but we were unable to associate its expression with the medial supporting cells. Distinguishing medial and lateral markers was hindered by relatively low cell numbers in the responding supporting cell group. We added a statement in the text to point out this limitation.

6. The authors also acknowledge that JAK/STAT signaling might be activated by other upstream signals (rather than IFN). Still, no evidence for the identity of these signals is given. It is stated that such signals would be “very challenging to identify.” I agree with this assessment and certainly sympathize with the author’s predicament. However, with the current data, it is impossible to make a firm correlation between IFN signaling, JAK/STAT activation and subsequent regeneration.

Yes, this is correct. We have removed any statements suggesting causal links.

7. The fact RUX treatment reduced expression of all IFN response genes except CCL4 suggests that this chemokine may be serving a different function than the other genes. One plausible role for CCL4 is a macrophage chemoattractant.

Yes, this is a good suggestion. We hope that our data will inspire subsequent studies and determining a role for CCL4 post-damage is certainly a possible new direction.

8. Both of the previous reviews noted that the data concerning CALB2 were quite distinct from the issues discussed above and, as a result, the manuscript appears to be unfocused. This is still the case.

We argued this point in the last review, and still stand by our rationale for including the CALB2 data because it presents a quite intriguing finding of high interest for the hair cell regeneration field.

In summary, I recommend that the authors provide some direct evidence of JAK/STAT activation and further show that the pathway is blocked by RUX.

We show that the damage-induced upregulation of the investigated genes is blocked by RUX (except for CCL4). We removed any statement for causal link of this pathway with regeneration and simply discuss our data. The lack of avian-specific antibodies that allow us to detect STATs (and P-STATS) in situ in supporting cells makes the activation experiment quite challenging. We argue, however, that there are many studies (which we cite) that link expression of the main group of genes that we identified and validated in our study (IFI6, IFIT5, OASL, RSAD2, LY6E) with JAK/STAT signaling.

Reviewer 2 Advance Summary and Potential Significance to Field...

I am satisfied with the answers to my queries/comments and the revisions to the manuscript. My main criticism was about the way the "link" between "responding SC" and "new HCs" was established/discussed and I thank the authors for clarifying their methodology. There is still room for debate about the significance of this link, and how are the "responding SC" contributing to the regenerating response... but overall the study provides extremely valuable insights into the molecular correlates of hair cell regeneration and I am looking forward to seeing the results of follow-up experiments on the specific roles of interferon signalling.

We thank the reviewer.
Three minor points:

- I think it is worth reminding the reader about the definition of "top ranking" genes (FDR<0.01

© 2022. Published by The Company of Biologists under the terms of the Creative Commons Attribution License (https://creativecommons.org/licenses/by/4.0/).

and Log2FC>2) in the section about common genes between responding SCs and new HCs (line 254). It was not clear to me that this was a standard for this paper, and in fact other sections rely on different thresholds (see line 104/Figure 1).

We added this threshold information in line 251. We were consistent with this threshold for all single cell RNA sequencing in this paper, and our recent baseline paper. Figure 1 is bulk RNA-sequencing data and FDR threshold was 0.05.

- The "*" representing statistical significance are now largely absent from Figure 5?

We removed the statistical analysis indicating * in cases where the comparison was not relevant to making specific points. The original plots were rather confusing with providing statistical data for all possible comparisons, some of which were irrelevant.

- In Figure 6F' it remains hard to see, at the individual cell nucleus level, the proportion of calretinin- positive cells that are also Edu+ (Sox2 signal is not helping). There's certainly some spatial overlap on the medial side, but not a strict correlation at the individual cell level. In fact, only a subpopulation of the calretinin+/Sox2+ SC are Edu+.. You could add a high mag view of samples stained for Edu/calretinin only to make this clearer and/or rephrase the statement line 265-66 (eg "...these supporting cells were in the proliferative phase of regeneration" changed to "... at least some of these supporting cells were in the proliferative phase...").

The goal of this figure is not to prove that every EdU+ cell is also CALB2 positive, but rather to demonstrate that CALB2 protein expression is coincident with the proliferative phase of regeneration. We clarify in the figure legend, "EdU was subcutaneously injected 2x into chickens at approximately 42 and 48 hours post-sisomicin". This is not sufficient to get every proliferating cell labeled with EdU. For this, we would need a line of experimentation similar to that in Figure 4, where EdU is injected every 6 hours starting earlier at 30 hours post-sisomicin.

Third decision letter

MS ID#: DEVELOP/2021/200113

MS TITLE: Avian auditory hair cell regeneration is accompanied by JAK/STAT-dependent expression of immune-related genes in supporting cells

AUTHORS: Amanda S Janesick, Mirko Scheibinger, Nesrine Benkafadar, Sakin Kirti, and Stefan Heller
ARTICLE TYPE: Research Article

I am happy to tell you that your manuscript has been accepted for publication in Development, pending our standard ethics checks.

Reviewer 1

Advance summary and potential significance to field

I believe that this was addressed in the previous reviews.

Comments for the author

The authors made a number of changes and additions in response to the last cycle of reviews. I feel that all important issues have been addressed and I have no further critiques of the current manuscript.

© 2022. Published by The Company of Biologists under the terms of the Creative Commons Attribution License (https://creativecommons.org/licenses/by/4.0/).
